# Exploration of the van der Waals Region of the NO *A* ^2^Σ^+^ + N_2_ *X* ^1^Σ_*g*_^+^ Collision Complex

**DOI:** 10.1021/acs.jpca.6c01405

**Published:** 2026-06-29

**Authors:** Alexandre De Matos Loja, Matthew L. Costen, Martin J. Paterson

**Affiliations:** Institute of Chemical Sciences, 3120 Heriot-Watt University, Edinburgh EH14 4AS, U.K.

## Abstract

The van der Waals (VdW) region of the potential energy surface (PES) of the collision complex of NO­(*A* ^2^Σ^+^) and N_2_(*X* ^1^Σ_*g*_^+^) was explored using explicitly correlated coupled-cluster methods. The Rydberg character of the NO­(*A* ^2^Σ^+^) state necessitated a sufficiently large and diffuse basis set. Basis set superposition error endemic to such systems was mitigated by the use of an explicitly correlated method in addition to a counterpoise correction, subsequent benchmarking was performed via both energetics and molecular properties such as the dipole and quadrupole moment. The method ultimately used was CCSD­(T)-F12a/t-aug-cc-pVTZ, with the full VdW PES constructed through a series of three-dimensional cuts exploring the space between key orientations. A broad main minimum well was observed surrounding the linear nitrogen-to-nitrogen (“LN”) orientation with a maximum depth of −239.51 cm^–1^ at an intermolecular separation of 4.2⁡Å . This new PES is useful for interpretation of recent molecular beam scattering experiments.

## Graphical abstract

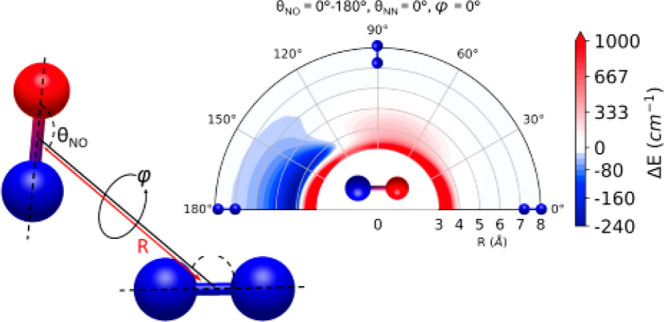

## 1 Introduction

Developments in the experimental study of stereodynamics and inelastic rotational energy transfer (RET) have been pushed by the introduction of experiments partnering velocity map imaging (VMI) with crossed molecular beam (CMB) experiments. Many such experiments focused initially on small-molecule collisions with inert monatomic gases, typically rare gases (Rg).^1–10^ This later progressed to a focus on the collision of larger inert gases, such as NO­(*X* ^2^Π) + D_2_, H_2_O + H_2_, ND_3_ + H_2_, and CH_3_ + H_2_.^11–14^ More recently, CMB-VMI experiments have begun to probe electronically excited molecules formed using pulsed laser excitation prior to collision, such as NO­(*A* ^2^Σ^+^).^15–21^ Collision experiments with excited state NO­(*A* ^2^Σ^+^) followed a similar trajectory, beginning with small Rg atoms (Ar and Ne) and then evolving into collisions with diatomics such as D_2_, N_2_, O_2_, and CO_2_.

Ab initio studies to produce accurate potential energy surfaces (PES) of collision complexes are often carried out to aid in the interpretation of such collision experiments, either directly or through further use in quantum-scattering calculations. Recently, we have experimentally explored the RET of NO­(*A* ^2^Σ^+^) in collisions with N_2_(*X* ^1^Σ_*g*_^+^) using CMB-VMI^21,22^ and although ab initio studies exist for this collision complex, all of these studies focus on specific regions or structures within the PES.^23–26^ A CMB scattering experiment necessarily involves all impact parameters and relative orientations and therefore fully explores the PES up to the repulsive wall or collision energy limit. A more holistic approach to calculating the PES of the NO­(*A* ^2^Σ^+^) and N_2_(*X* ^1^Σ_*g*_^+^) collision complex, which looks at all possible orientations, would therefore be beneficial toward the discussion and interpretation of scattering experiments on the collision complex and so we look to provide that here.

Although we have only explored the NO­(*A* ^2^Σ^+^) + N_2_(*X* ^1^Σ_*g*_^+^) collision complex experimentally up until now, we have previously explored ab initio studies into NO­(*A* ^2^Σ^+^) collision complexes with O_2_(*X* ^3^Σ_*g*_^–^) and CO_2_(*X* ^1^Σ_*g*_^+^) collision partners.^27–29^ The work carried out on NO­(*A* ^2^Σ^+^) + O_2_(*X* ^3^Σ_*g*_^–^) laid much of the groundwork for the study on NO­(*A* ^2^Σ^+^) + CO_2_(*X* ^1^Σ_*g*_^+^) as well as this one through extensive benchmarking. A focus was placed particularly on identifying the ideal basis set and methodology to accurately calculate the Rydberg character of the 4σ orbital of the excited *A* state. This was carried out by calculating molecular properties (i.e., di/quadrupole moments and polarizability) of the participating molecules as a measure of the efficacy of the method. The Dunning correlation-consistent family of basis sets was employed due to their hierarchical nature to allow for systematic testing of basis set sizes.^30^ An “even-tempering” approach was also used to progressively add additional diffuse functions following the formula introduced by Glendening et al.^31^ A crucial need for, at minimum, a double augmented level (e.g., d-aug-cc-pVnZ) of basis set to accurately calculate the molecular properties of the (*A* ^2^Σ^+^) state of NO was observed, leading to the use of the aug-cc-pVTZ-333 basis set (the “333” specifying the number of additional diffuse functions, in this instance equivalent to a triply augmented level) in subsequent calculations. The same level of benchmarking was carried out for the partner molecule, though O_2_ in the ground state was less sensitive to basis set changes. A reduced form of the same benchmarking was then repeated in the study of the NO­(*A* ^2^Σ^+^) + CO_2_(*X* ^1^Σ_*g*_^+^) collision complex and was also replicated for our work here on the NO­(*A* ^2^Σ^+^) + N_2_(*X* ^1^Σ_*g*_^+^) complex, later discussed in Sections 3.2.

For benchmarking the molecular properties of the partner molecule N_2_(*X* ^1^Σ_*g*_^+^), the quadrupole moment is used (*Q*_*zz*_), thus accurate experimental or calculated values of *Q*_*zz*_ is needed. An experimental *Q*_*zz*_ value of −4.65 ± 0.08 × 10^–40^ cm^2^ was obtained by Graham et al., though, this value disagrees with many values obtained through ab initio calculations.^32–36^ One of the attempts to calculate the *Q*_*zz*_ of N_2_(*X* ^1^Σ_*g*_^+^) was performed by Halkier et al. obtaining a *Q*_*zz*_ of −4.93 ± 0.03 × 10^–40^ cm^2^ using (CCSD­(T)) alongside a sufficiently diffuse basis set with extrapolation to the complete basis set (CBS) limit. Given the disparity, Halkier et al. challenged the derivation by Graham et al., looking to rederive the value of *Q*_*zz*_ using the experimentally obtained effective quadrupole moment *Q*_eff_ from Graham et al.^37^ The *Q*_*zz*_ is obtained from the following equation^32^

1 $$Q=Q_{eff}-\frac{15bkT}{2\Delta \alpha }$$

The problem was highlighted to be the *b* term of the equation, which Graham et al.^32^ obtained using a number of hyperpolarizability terms calculated through ab initio techniques (such as described in ref 38). Halkier et al.^37^ recalculated the necessary hyperpolarizability terms, providing a new value for *b* with which to derive *Q*_*zz*_ from the experimentally obtained *Q*_eff_ of Graham et al. giving a new *Q*_*zz*_ of −5.01 ± 0.08 × 10^–40^ cm^2^. This value was far more consistent with values obtained from various other ab initio techniques.^34,35,39^

A prominent issue to consider when performing calculations on systems of weakly bound or interacting dimers or complexes such as the NO­(*A* ^2^Σ^+^) + N_2_(*X* ^1^Σ_*g*_^+^) is the impact of basis set superposition error (BSSE). The most direct solution would be to attempt to extrapolate to the complete basis set (CBS) limit. However, this method requires performing multiple calculations of increasing basis set size for any given point of the PES. Ultimately as a large number of points are calculated along the PES, extrapolating to the CBS limit becomes prohibitive. The use of a counterpoise (CP) correction offers an alternate remedy, looking to minimize BSSE. The efficacy of this method is sometimes disputed^40,41^ but it remains by far the most popular route to address BSSE. van Duijneveldt et al. argues that the rigor of a CP correction is due to the confusion of BSSE with basis set incompleteness error (BSIE) and configuration set superposition error (CSSE).^42^ They discuss that a CP correction will always entirely remove the BSSE, but only relevant to a given method and the basis set used. A CP correction does not tackle BSIE or CSSE and therefore cannot provide an improved result past the inherent limitations of the method and basis set. The use of a CP correction, therefore still necessitates a sufficiently good basis set to be used, and only through reaching the CBS limit will the BSIE be removed and through a full configuration interaction (FCI) result will the CSSE be removed. The straightforwardness of carrying out a CP correction has resulted in a large body of benchmarking to explore its efficacy on reducing BSSE.^43,44^ One trend is clear from these benchmarks: as the size of the basis set increases, the disparity between results obtained using the non-CP and CP approaches shrinks, with the two values converging on the true value in the CBS limit.

In recent years, explicitly correlated methods, such as the coupled cluster variant: “CC-F12”, have been developed, introducing terms relating to the interelectronic distance to improve the quality of the wave function.^45–47^ The results are generally a wave function that exhibits accuracy in line with an ζ ≈ *n* + 2 larger-sized basis set than the one used.^48^ It was then discovered that this also led to an apparent reduction in BSSE.^41,45,48^ However, in contrast to the CP approach, an explicitly correlated method does more to tackle BSIE than BSSE directly by effectively providing a result equivalent to a larger basis set. If the effective increase to the basis set size is sufficient it can appear to completely remove BSSE, though in reality it does not completely remove either BSIE or BSSE. This is unlike the CP correction which by design entirely removes BSSE relative to the basis set and method. As such, particularly with smaller basis sets, or where there is still a large separation between a CP and non-CP corrected result, it is advisable to combine a CP correction with an explicitly correlated method, as supported from benchmarking carried out by McMahon and Lane.^45^

As mentioned, the NO­(*A* ^2^Σ^+^) + N_2_(*X* ^1^Σ_*g*_^+^) complex has been previously explored using ab initio methods, typically in support of experimental work. As a result, these studies focus around electronic state transitions and photodissociation dynamics, so look to calculate only specific structures or portions of the larger PES.^21,23–26^ There are two notable studies by Lozeille et al.^23^ and Guardado et al.,^25^ who both establish the global minimum energy structure of the NO­(*A* ^2^Σ^+^) + N_2_(*X* ^1^Σ_*g*_^+^) complex and approximated the binding energy of the *A* state. Both studies found the global minimum energy structure to be a linear nitrogen-to-nitrogen facing orientation with an intermolecular distance (N-to-N) of ≈3.2 Å. Guardado et al.,^25^ using electron-attachment equation-of-motion coupled-cluster singles and doubles (EOM-EA-CCSD) and the d-aug-cc-pVTZ basis set, obtained a binding energy of 335.02 cm^–1^. Lozeille et al. obtained a lower binding energy of 231 cm^–1^ using restricted coupled cluster singles doubles with perturbative triplets (RCCSD­(T)) using the aug-cc-pVTZ­(Ryd-2) basis set (roughly equivalent in size to the one used by Guardado et al.). The difference can largely be attributed to the additional CBS limit extrapolation performed by Lozeille et al. In the discussion of the binding energy of the *A* state, Lozeille et al. also explored this experimentally reporting a much lower value of 143 ± 0.5 cm^–1^. They attributed the difference largely to zero-point corrections but could not fully rationalize the large discrepancy.

## 2 Computational Details

All calculations were performed using version 2023.2 of the MOLPRO package.^49–51^ In all instances, a NO­(*A* ^2^Σ^+^) state reference wave function was generated using restricted Hartree–Fock (RHF/aug-cc-pVDZ) by the rotation of the highest occupied MO to the 4σ orbital to produce the desired electronic (NO­(*A* ^2^Σ^+^)) state. This was then followed up by a “step-up” RHF calculation using the larger, desired basis set before reading this wave function into the desired final calculation. The “step-up” RHF calculation was introduced to prevent the wave function collapsing back to the ground state, when attempting to read in the wave function for the CC method after the orbital rotation. The correct state was identified using the Molden visualization software to visually confirm the 4σ Rydberg orbital.^52,53^ Later calculations of the full PES were performed starting from the largest intermolecular distance wherein the 4σ orbital is best defined, due to minimal interactions with the N_2_ molecule, with calculations of the shorter intermolecular distances performed reading the wave function from the previous point of the surface to ensure the consistency of the electronic state. Corroboration that the correct *A* state was being calculated and maintained was done through the visual confirmation of the orbitals at convergence.

The intermolecular distance (*R*) covered in the PES was set within the range of 15 Å → 3.2 Å, adapting from our past study on the van der Waals (VdW) region of the NO­(*A* ^2^Σ^+^) + O_2_(*X* ^3^Σ_*g*_^–^) collision complex.^29^ The step size of *R* was increased from 0.1 Å to 2 Å as *R* increased given the relative lack of features in the PES at the larger intermolecular distances. The outer limit of 15 Å is arbitrarily set to a distance of sufficient separation such that interactions between the molecules is minimal to none. The inner limit of *R* = 3.2 Å employed was kept similar to the value of 3.4 Å used in the study of the NO­(*A* ^2^Σ^+^) + O_2_(*X* ^3^Σ_*g*_^–^) collision complex. This intermolecular distance had been decided in the past study based on the growing involvement of multireference character in the wave function as *R* decreases. The multireference character was estimated using the T1 and D1 diagnostics with a T1 value above 0.02 being considered a sign of growing multireference character and therefore set as a soft-limit to indicate a single-reference treatment is ineffective.^54^ The T1 diagnostic was similarly calculated here for the NO­(*A* ^2^Σ^+^) + N_2_(*X* ^1^Σ_*g*_^+^) to confirm the ideal *R* range and is later discussed in Section 3.

A single-reference approach was taken to calculate the PES, opting to use the explicitly correlated variant of coupled-cluster singles and doubles with perturbative triplets (CCSD­(T)-F12a).^46,47,55–61^ Both our previous calculations on the surfaces for the NO­(*A* ^2^Σ^+^) + O_2_(*X* ^3^Σ_*g*_^–^) and NO­(*A* ^2^Σ^+^) + CO_2_(*X* ^1^Σ_*g*_^+^) complexes, as well as the calculations performed by Klos et al. on NO­(*A* ^2^Σ^+^)-Rg complexes have found convergence to the *A* state using CCSD­(T) to be possible and stable, with no variational collapse to the ground state.^62^ The ability to use CCSD­(T) was a benefit, given the easier setup and size consistent nature of the method compared to complete active space self-consistent field (CASSCF) and multireference configuration interaction (MRCI) methods. The explicitly correlated version of coupled-cluster was employed to further improve the quality of the wave function with minimal added computational cost. In particular, the F12a variation was used as it has been noted to provide better results for triple-ζ basis sets.^49^ The augmented correlation consistent Dunning basis sets (aug-cc-pV*n*Z, *n* = *d*/*t*/*q*/5) have been used, due to their hierarchical nature for ease of benchmarking, size adjustment, and addition of extra diffuse functions.^30^

### 2.1 Coordinate System

The Jacobi coordinate system was introduced in the study of the NO­(*A* ^2^Σ^+^) + O_2_(*X* ^3^Σ_*g*_^–^) collision complex and continued to be used for the NO­(*A* ^2^Σ^+^) + CO_2_(*X* ^1^Σ_*g*_^+^) collision complexes as an efficient means to calculate and describe the PES.^27–29^ The coordinate system breaks down the surface into the variables: *R*, θ_NO_, θ_NN_, and ϕ. The length *R*, describes the intermolecular distance between the centers of masses of both molecules (which is referred to here as just the intermolecular distance for the sake of brevity). θ_NO_ and θ_NN_ define the angle created at the point the *R* vector intersects the center of mass of the respective molecular bond axis. Finally, the ϕ angle describes the dihedral angle created between the two molecules. These variables are illustrated in Figure 1. The only fixed variables are the bond lengths of the two molecules to reduce the overall complexity of the surface and number of calculations needed. Bond lengths are fixed to their equilibrium length for their given state, so *R*_NO_ = 1.0643 Å and *R*_NN_ = 1.098 Å.^29,33^

**Figure 1:**
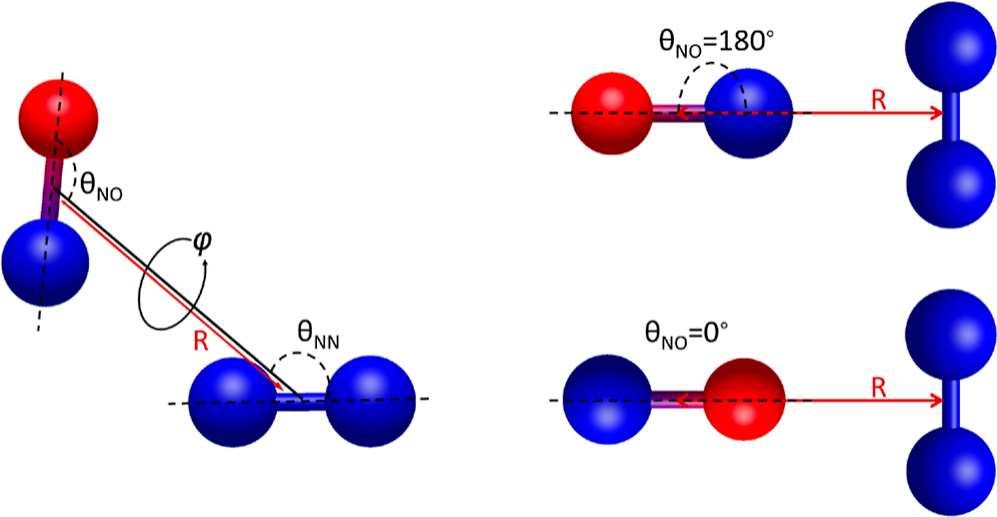
Illustration of the Jacobi coordinates applied to the NO­(*A* ^2^Σ^+^) + N_2_(*X* ^1^Σ_*g*_^+^) system used to define the PES of the collision complex (LHS). Illustration defining the θ_NO_ angle, depending on which atom of NO is facing N_2_ (RHS).

### 2.2 Molecular Properties

We sought to perform similar benchmarking on the NO­(*A* ^2^Σ^+^) + N_2_(*X* ^1^Σ_*g*_^+^) collision complex as was performed on the NO­(*A* ^2^Σ^+^) + O_2_(*X* ^3^Σ_*g*_^–^) collision complex.^29^ This involved calculating static electric properties of both molecules in the form of the static dipole/quadrupole moment (for NO­(*A* ^2^Σ^+^) and N_2_(*X* ^1^Σ_*g*_^+^), respectively) as well as their polarizability using the finite field method. Each molecule was calculated in the presence of an external electric field taking the first and second derivatives of the energy to obtain the static dipole/quadrupole moment and polarizability, respectively. The details of the calculations closely follow those outlined in ref 29. The only changes were a tighter convergence to the CC portion of the calculation to 1 × 10^–10^ au.

### 2.3 Treatment of BSSE

Additional benchmarking was carried out with the focus on BSSE reduction as well as the efficacy of the explicitly correlated method and CP correction. Due to the limitations of the software package to perform the finite field method in conjunction with the explicitly correlated methods, the focus of the benchmarking was shifted to describing the properties of a known minimum energy well. Using the observed global minimum energy in past studies of the NO­(*A* ^2^Σ^+^) + N_2_(*X* ^1^Σ_*g*_^+^) collision complex, located at the LN orientation, a one-dimensional cut was performed along the *R* values for the LN orientation.^21,23,25^ The cuts were calculated using both the explicitly correlated CCSD­(T)-F12a and CCSD­(T) methods for a handful of basis sets.

The CCSD­(T)-F12 calculations require auxiliary basis sets in the form of the density fitting (DF) and resolution of the identity (RI) basis sets. Typically, these are included within the software package for the core basis set, however, as larger basis sets with higher levels of augmentation (i.e., up to the t-aug-basis set) were used, the complementary DF and RI basis sets were unavailable. Thus, a standard basis was expanded (e.g., aug-cc-pVTZ to t-aug-cc-pVTZ) in an even-tempered manner, following α_+1_ = α^2^/α_–1_.^31^

## 3 Results and Discussion

### 3.1 Static Electric Properties and BSSE Mitigation

The dipole moments for NO in the (*X* ^2^Π) and (*A* ^2^Σ^+^) states, using different basis sets, are presented in Tables 2 and 3, respectively. Experimental results as well as values from NO­(*A* ^2^Σ^+^) + O_2_(*X* ^3^Σ_*g*_^–^)/CO_2_(*X* ^1^Σ_*g*_^+^) are also provided for comparison. The general trend seen is that an increase in the size of the basis set (through an increased ζ value or the addition of extra diffuse functions) improves the resulting value (dipole or quadrupole), such that it approaches the experimental value. In all cases, the initial increase of diffuse functions, from nonaugmented to singly augmented and then doubly augmented, has the largest impact on the resulting molecular property, compared to increasing the ζ value of the basis set. For example, the difference between the calculated value and experimental value of μ_*z*_^α^ for NO­(*X* ^2^Π) is 0.02 D using cc-pVQZ but only 0.005 D with aug-cc-pVDZ.

**Table 1:** Names for the Key Orientations (with Respective Acronyms) Observed in the NO­(*A* ^2^Σ^+^) + N_2_(*X* ^1^Σ_*g*_^+^) Collision Complex PES along with the Associated Jacobi Coordinates Reported in Degrees (°)

orientation name	θ_NO_	θ_NN_	ϕ
linear-oxygen (LO)	0	0/180	-
Hammer (H)	90	0/180	-
linear-nitrogen (LN)	180	0/180	-
T-shape-nitrogen (TN)	180	90	-
parallel (P)	90	90	0
T-shape-oxygen (TO)	0	90	-
cross (C)	90	90	90

**Table 2:** Dipole Moments (μ_*z*_) and Polarizability (α) for NO­(*X* ^2^Π) Calculated Using CCSD­(T) and the Finite Field Method, Reported in Units of Debye and *a*_0_^3^, Respectively^a^

basis set	μ_*z*_^*a*^	α_*xx*_	α_*yy*_	α_*zz*_	⟨α⟩
cc-pVDZ	–0.101	5.00	4.60	11.80	7.13
aug-cc-pVDZ	–0.153	8.60	9.60	14.90	11.03
d-aug-cc-pVDZ	–0.154	9.20	10.40	15.40	11.67
t-aug-cc-pVDZ	–0.156	9.40	10.40	15.40	11.73
cc-pVTZ	–0.135	6.40	6.30	12.90	8.53
aug-cc-pVTZ	–0.159	9.20	9.80	15.10	11.37
aug-cc-pVTZ^*a*^	–0.167	9.12	9.95	15.04	11.37
d-aug-cc-pVTZ	–0.159	9.30	10.10	15.30	11.53
d-aug-cc-pVTZ^*a*^	–0.167	9.30	10.18	15.21	11.57
t-aug-cc-pVTZ	–0.159	9.40	10.10	15.20	11.57
t-aug-cc-pVTZ^*b*^	–0.167				
cc-pVQZ	–0.138	7.60	7.70	13.70	9.67
aug-cc-pVQZ	–0.153	9.30	10.00	15.10	11.47
aug-cc-pVQZ^*a*^	–0.161	9.21	10.04	15.04	11.43
d-aug-cc-pVQZ	–0.152	9.40	10.20	15.30	11.63
d-aug-cc-pVQZ^a^	–0.160	9.26	10.11	15.09	11.49
t-aug-cc-pVQZ	–0.152	9.40	10.20	15.20	11.60
exp^*c*^	–0.158 ± 0.006				11.70 ± 0.27
exp^*d*^					11.52 ± 0.01

a The isotropic polarizability (⟨α⟩) was calculated from the average trace values: αxx, αyy, and αzz. Comparative values provided from previously reported ab initio (a),^29^ (b),^28^ and experimental results (c),^63,64^ (d).^65^ Positive values are defined as N + O–.

**Table 3:** Dipole Moments (μ_*z*_) and Polarizability (α) for NO­(*A* ^2^Σ^+^) Calculated Using CCSD­(T) and the Finite Field Method, Reported in Units of Debye and *a*_0_^3^, Respectively^a^

basis set	μ_*z*_^*a*^	α_*xx*/*yy*_	α_*zz*_	⟨α⟩
cc-pVDZ	0.301	4.8	10.3	6.63
aug-cc-pVDZ	2.417	244.6	142.60	210.60
d-aug-cc-pVDZ	1.112	616.8	417.90	550.77
t-aug-cc-pVDZ	1.119	621.8	419.70	554.43
cc-pVTZ	0.345	6.4	11.30	8.07
aug-cc-pVTZ	2.552	306.7	206.70	273.367
aug-cc-pVTZ^*a*^	2.54	293.3	218.9	268.5
d-aug-cc-pVTZ	1.070	605.90	412.90	541.57
d-aug-cc-pVTZ^*a*^	1.00	582.1	415.8	526.7
t-aug-cc-pVTZ	1.075	614.9	412.60	547.47
t-aug-cc-pVTZ^*b*^	1.09			
cc-pVQZ	0.352	7.60	12.40	9.200
aug-cc-pVQZ	2.470	356.0	261.90	324.63
aug-cc-pVQZ^*a*^	2.42	340.2	275.6	318.7
d-aug-cc-pVQZ	1.059	603.70	410.50	539.30
d-aug-cc-pVQZ^*a*^	0.99	580.8	416.9	526.2
t-aug-cc-pVQZ	1.063	613.00	410.30	545.43
exp^*c*^	1.08 ± 0.04			

a The isotropic polarizability (⟨α⟩) was calculated from the average trace values: α_*xx*_, α_*yy*_, and α_*zz*_. Comparative values provided from previously reported ab initio (a),^29^ (b),^28^ and experimental results (c).^66^ Positive values are defined as N^+^O^–^.

The disparity between the impact on the resulting values between changing the ζ level of the basis set and the cardinal number of the augmentation of the basis set is more severe for the properties of NO­(*A* ^2^Σ^+^). Increasing the ζ value of the basis set still results in a convergence toward the reported experimental value, but this is very minor in comparison to increasing the diffuseness of the basis set. For example, the difference in the calculated and experimental value of μ_*z*_^α^ using the cc-pVDZ basis set is equal to 0.779 ± 0.04 D, with only a slight improvement to 0.728 ± 0.04 D using the cc-pVQZ basis set. The initial increase to a singly augmented basis set leads to a large over estimation, however, increasing this to a doubly augmented level sees another substantial change, but much closer to the experimental value. This is similar to the findings from the benchmarking performed on the NO­(*A* ^2^Σ^+^) + O_2_(*X* ^3^Σ_*g*_^–^) collision complex, which noted that at least a doubly augmented level of basis set is required. For example, using the aug-cc-pVQZ basis set results in a difference of 1.39 ± 0.04 D to the experimental value, which is reduced to 0.021 ± 0.04 D using the d-aug-cc-pVQZ basis set. This is anticipated given the Rydberg character of NO­(*A* ^2^Σ^+^). The value within experimental error bounds is reached with a t-aug-cc-pVQZ level of basis.

The *Q*_*zz*_ for N_2_(*X* ^1^Σ_*g*_^+^) using the different basis sets are presented in Table 4, again with comparative values provided. Regardless of the method used, our reported value, more closely agreed with the re-evaluated value of 5.01 ± 0.08 × 10^–40^ Cm^2^ by Halkier et al. rather than the value of −4.65 ± 0.08 × 10^–40^ cm^2^ of Graham et al.^32,37^ The general trend is still that, as the size of the basis set used increases, regardless of increasing diffuseness or ζ size, the reported *Q*_*zz*_ is closer to the value reported by Halkier et al. The biggest difference is seen by changing the ζ value of the basis set, for example, a difference of only 0.001 ± 0.08 × 10^–40^ cm^2^ is observed using d-aug-cc-pVQZ from the results of Halkier et al. However, this value is well within the experimental error, and the increase in computational cost to move to a quadruple-ζ basis set is substantial enough that it is more effective to remain at a triple-ζ level.

**Table 4:** Quadrupole Moments (*Q*_*zz*_/*Q*_*xx*/*yy*_) for N_2_(*X* ^1^Σ_*g*_^+^) Calculated Using CCSD­(T) and the Finite Field Method, Reported in Units of × 10^–40^ cm^2^ ^a^

basis set	*Q*_*zz*_	*Q*_*xx*/*yy*_
cc-pVDZ	–6.052	3.026
aug-cc-pVDZ	–4.986	2.492
d-aug-cc-pVDZ	–5.122	2.560
t-aug-cc-pVDZ	–5.131	2.564
cc-pVTZ	–5.522	2.761
aug-cc-pVTZ	–5.015	2.506
d-aug-cc-pVTZ	–5.041	2.519
t-aug-cc-pVTZ	–5.049	2.523
cc-pVQZ	–5.271	2.635
aug-cc-pVQZ	–4.999	2.499
d-aug-cc-pVQZ	–5.011	2.504
t-aug-cc-pVQZ	–5.014	2.506
exp^*a*^	–4.65 ± 0.08	2.320
exp^*b*^	–5.01 ± 0.08	
ref^*c*^	–4.93 ± 0.03	

a Comparative values provided from previously reported experimental (a),^32,33^ (b),^37^ and ab initio results (c).^37^

The secondary set of preliminary calculations surrounding the efficacy of describing the known minimum energy well at the “LN” orientation are presented in Table 5. Energies presented here are calculated as the difference in energy between the minimum point and the energy at maximum separation (*R* = 15 Å) for the same method. A clear convergence of both the location (*R*_min_) and energy (Δ*E*) of the minimum well is observed as the basis set size increases and the different considerations for BSSE (explicitly correlated method and CP correction) are implemented. The difference between the non-CP and CP corrected energy minimum are presented here as a measure of the quality of the surface. If we take the discussion from section 1, Duijneveldt et al.^42^ argue that the CP correction by construction does in-fact remove all BSSE, but differences to the “true” value arise in the form of the BSIE and CSSE. It is likely for this reason that the benchmarking carried out by Brauer et al., Gray et al., and Burns et al. all show a convergence, albeit nonuniform, when comparing the non-CP and CP results over increasing basis set sizes.

**Table 5:** Resulting Minimum Energy Well Locations (*R*) and Depths (Δ*E*) along the PES Cut Made between *R* = 15 Å → 3.2 Å on the “LN” Orientation for the NO­(*A* ^2^Σ^+^) + N_2_(*X* ^1^Σ_*g*_^+^)­^a^

	non-CP corrected		CP corrected		difference
basis set	*R*_min_ (Å)	Δ*E* (−cm^–1^)	*R*_vdW_ (Å)	Δ*E* (−cm^–1^)	Δ*E* (cm^–1^)
CCSD(T)/aug	4.2	1247.5	4.3	181.2	1066.3
CCSD(T)/d-aug	4.2	430.3	4.3	229.2	201.1
CCSD(T)/t-aug	4.2	424.6	4.2	231.8	192.8
F12/aug	4.2	764.3	4.3	155.1	609.2
F12/d-aug	4.2	272.4	4.2	238.8	33.6
F12/t-aug	4.2	271.5	4.2	239.4	32.1

a Step size for *R*, outlined in Section 3 and the Supporting Information, though, a step size of *R* = 0.1 Å was used surrounding the reported values. The “-cc-pVTZ” portion of the basis set names has been omitted for brevity. Results are reported with a (RHS) and without (center) a CP correction with the resulting difference in Δ*E* on the LHS.

As with the results of the calculated molecular properties, increasing the diffuse nature of the basis set above the singly augmented level provides the most significant improvement. This is clear in both the standard CCSD­(T) and the explicitly correlated method which report a drop in non-CP and CP differences of 865.5 cm^–1^ and 575.6 cm^–1^, respectively, when moving from a singly augmented to a double-augmented basis set. Similar to the findings of the molecular property calculations, there are diminishing returns when increasing the diffuse nature of the basis set to a triply augmented level. As for the efficacy of the explicitly correlated method, we can see that there is a definite improvement as in all cases the CP to non-CP difference is far more converged than the comparative standard CCSD­(T) results. For example, the CP to non-CP difference seen using CCSD­(T) and CCSD­(T)-F12 with the same t-aug-cc-pVTZ is an almost 6-fold reduction from 192.8 cm^–1^ to 32.1 cm^–1^, respectively. This degree of improvement especially highlights the benefits of the explicitly correlated method, as the additional computational cost is much lower than further increasing the size of the basis set to a ζ = 4 quality basis set. Though moving toward a pVQZ level of basis set would likely see a further convergence in the minimum energy well, it is unlikely to be a large improvement from a Δ*E*_diff_ of 32.1 cm^–1^ already obtained using t-aug-cc-pVTZ, for a substantial increase in computational cost.

### 3.2 Full Potential Energy Surface

The PES of the NO­(*A* ^2^Σ^+^) + N_2_(*X* ^1^Σ_*g*_^+^) complex was calculated using CCSD­(T)-F12/t-aug-cc-pVTZ over 23,777 points. All energies in the PES are reported relative to the energy at the maximum point of separation (15 Å) which has been set to Δ*E* = 0. The overall PES was segmented into a series of 3-dimensional cuts covering 180°of rotation of a single angle with all other angles fixed. The surfaces involving the orientational space and key orientations highlighted in Figure 2 and Table 1 are presented in Figures 3–5. An angular step size of 5° was used covering 31 values of *R* as previously outlined in Section 2. Additional surfaces within the orientational space proposed in Figure 2 are presented in the Supporting Information, further expanding the PES for use in scattering calculations.

**Figure 2:**
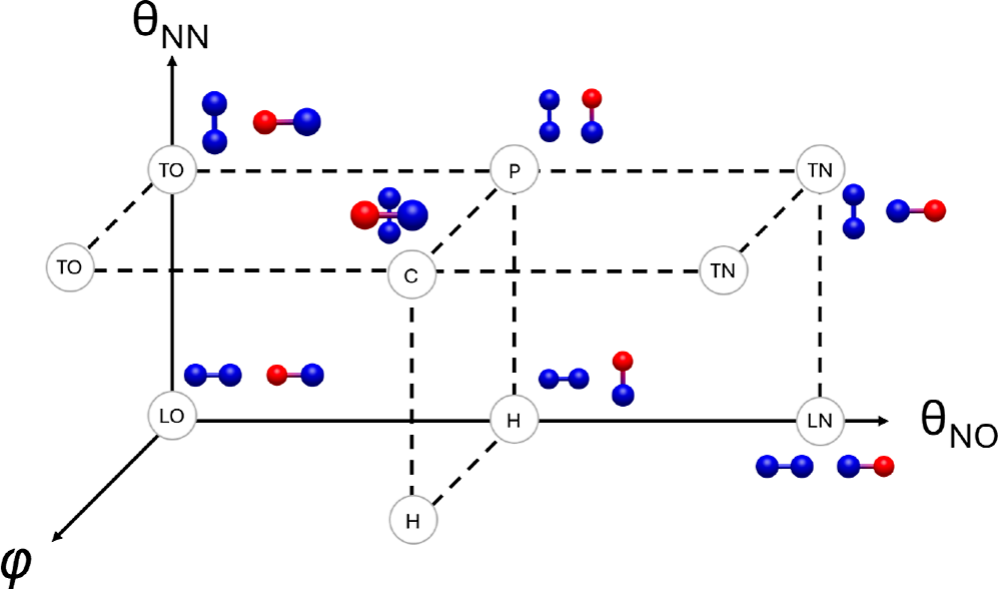
Illustration of the orientational space investigated between the highlighted orientations given in Table 1. Visualizations of the different orientations are also provided alongside their location in the orientational space (dashed lines highlight the three-dimensional cuts of the PES presented in Section 3).

**Figure 3:**
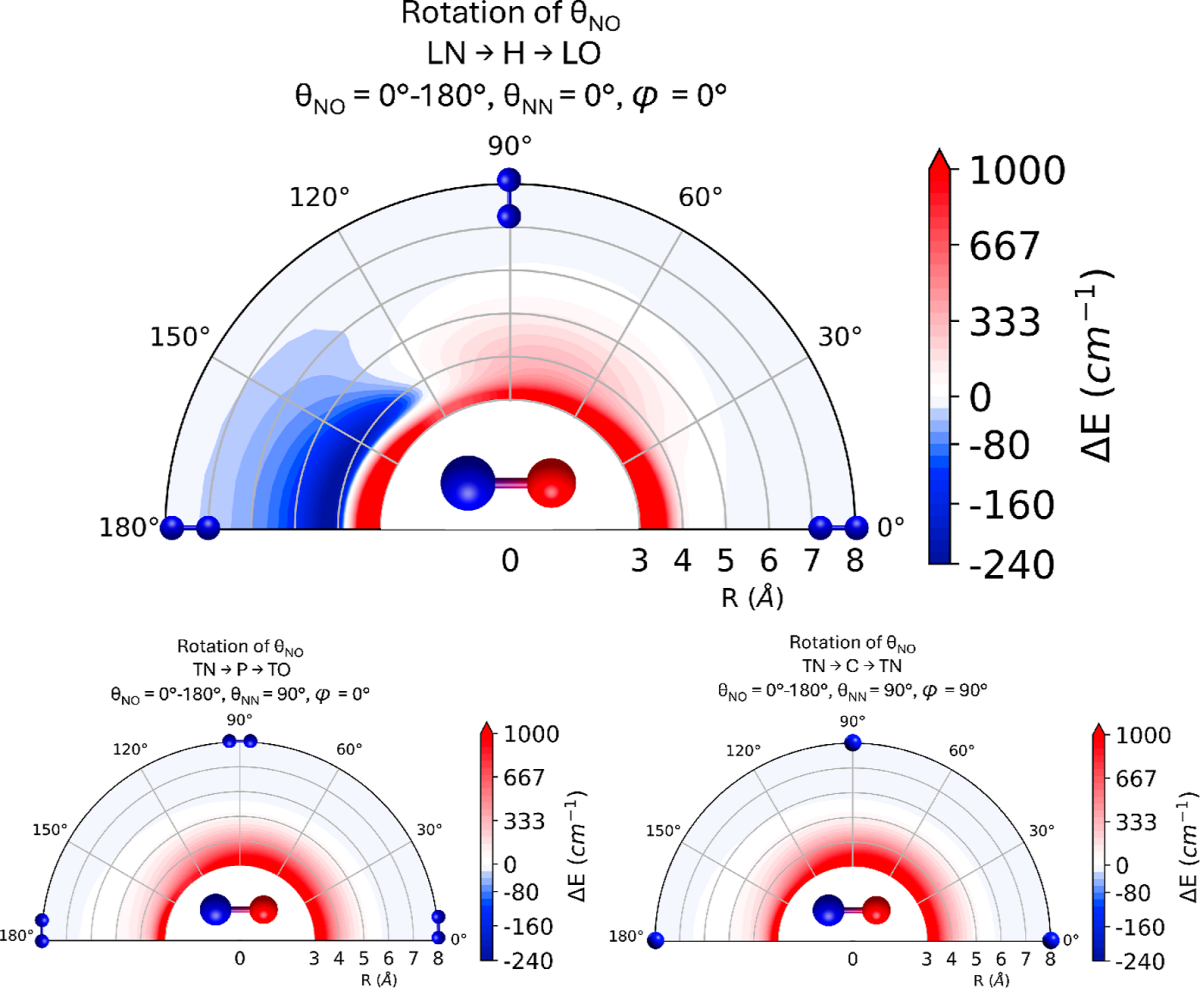
VdW PES cuts for the rotation of θ_NO_ in the NO­(*A* ^2^Σ^+^) + N_2_(*X* ^1^Σ_*g*_^+^) collision complex (CCSD­(T)-F12a/t-aug-cc-pVTZ, CP corrected). Energies reported relative to the maximum separation of the complex (15 Å). The repulsive wall of the PES at short *R* values has been “flattened” past a cutoff of 1000 cm^–1^ and artificially extended to *R* = 3 Å, though no points past *R* = 3.2 Å were calculated.

**Figure 4:**
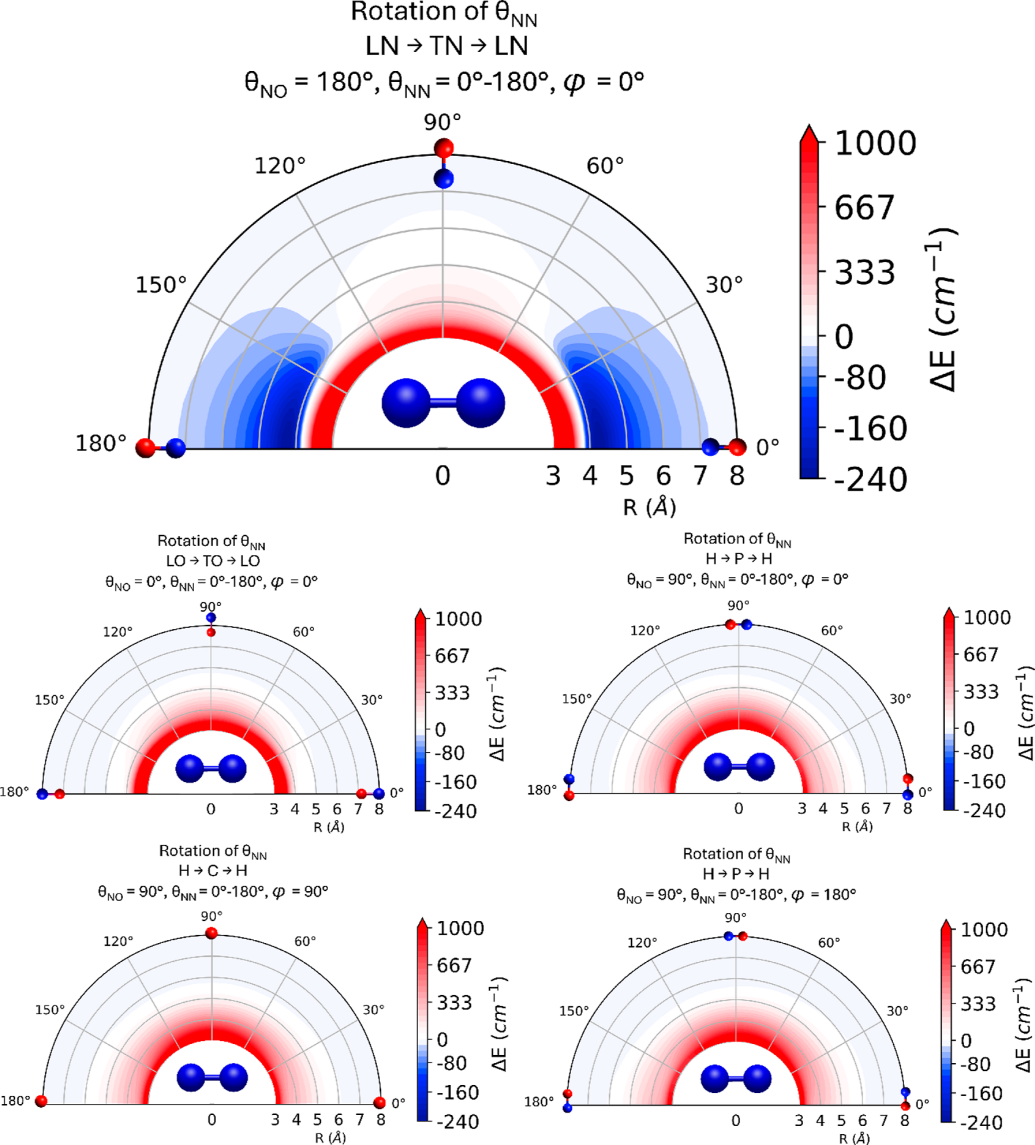
VdW PES cuts for the rotation of θ_NN_ in the NO­(*A* ^2^Σ^+^) + N_2_(*X* ^1^Σ_*g*_^+^) collision complex (CCSD­(T)-F12a/t-aug-cc-pVTZ, CP corrected). Energies reported relative to the maximum separation of the complex (15 Å). The repulsive wall of the PES at short *R* values has been “flattened” past a cutoff of 1000 cm^–1^ and artificially extended to *R* = 3 Å, though no points past *R* = 3.2 Å were calculated.

**Figure 5:**
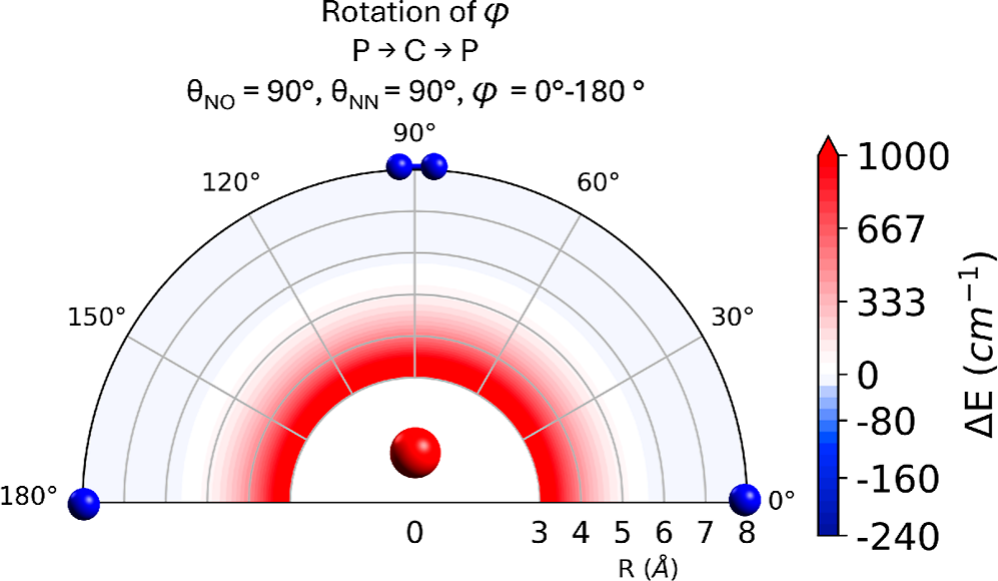
VdW PES cut for the rotation of ϕ in the NO­(*A* ^2^Σ^+^) + N_2_(*X* ^1^Σ_*g*_^+^) collision complex (CCSD­(T)-F12a/t-aug-cc-pVTZ, CP corrected). Energies reported relative to the maximum separation of the complex (15 Å). The repulsive wall of the PES at short *R* values has been “flattened” past a cutoff of 1000 cm^–1^ and artificially extended to *R* = 3 Å, though no points past *R* = 3.2 Å were calculated.

As mentioned in Section 2, the T1 diagnostic was also calculated for each point^54^ to attempt to approximate the involvement of multireference character. A full breakdown of the T1 diagnostics obtained for the surface are presented in the Supporting Information. The key results show a large portion of the PES has a T1 value above 0.02, 14.8% is below 0.02 and 47.8% is below 0.021. However, the values obtained for the area surrounding the discovered minimum energy wells in the PES were observed to be below the 0.02 threshold. The majority of the higher T1 value points appear at closer *R* values of 4.1 Å → 3.2 Å as is to be expected. The lowest identified minimum well is observed around the “LN” orientation, reaching a maximum depth of Δ*E* = −239.51 cm^–1^ at R=4.2⁡Å , θ_NO_ = 180°, θ_NN_ = 0°, and ϕ = 0°. The well is characteristically broad, extending to Θ_NO_ ≈ 135° or R≈7⁡Å before Δ*E* begins to sharply increase, though only reaching Δ*E* ≈ 0 at around R = 11 Å. The orientation and general parameters match the optimized structure of Lozeille et al. and Guardado et al.^21,23,25^ For a more direct comparison to the reported minimum energy structures of Lozeille et al. and Guardado et al., we converted the intermolecular distance to the distance between the two closest nitrogen atoms, as they have done. This gave us an $R_{N\cdot \cdot \cdot N}=3.1\pm 0.1Å$ at the global minimum energy, which is in excellent agreement with the reported values of $R_{N\cdot \cdot \cdot N}=3.2268Å$ and $R_{N\cdot \cdot \cdot N}=3.19Å$ by Lozeille et al. and Guardado et al., respectively.

Additional notable characteristics of the PES include a shallow attractive band along almost all orientations occurring at *R* ≈ 5.8– 9⁡Å and reaching a depth of Δ*E* ≈ −20 cm^–1^. For many cuts of the surface, this band is isotropic. Breaks in this band occur at the global minimum, the opposite linear LO orientation, at the TN orientation and the H orientation. The LO orientation (the opposing linear orientation to the global minimum) appears to be another, smaller, minimum energy well extending the previously mentioned attractive isotropic band to *R* ≈ 5.4– 11⁡Å . A maximum depth of Δ*E* = −25.21 cm^–1^ is observed at R=6.5⁡Å , θ_NO_ = 0°, θ_NN_ = 0°, and ϕ = 0°. The TN orientation (most clearly observed in Section 3) sees a break in the minimum energy band and can be considered entirely repulsive along all values of *R*. Along this orientation, the lowest value of Δ*E* reaches −1.26 cm^–1^ at *R* = 7, θ_NO_ = 180°, θ_NN_ = 90°, and ϕ = 0°. The H orientation, similar to the TN orientation (most clearly observed in Section 2), also shows a region of high repulsion, although with less intensity compared to the TN orientation. The lowest value of Δ*E* reached, along the H orientation, was −11.62 cm^–1^ at *R* = 7, θ_NO_ = 90°, θ_NN_ = 0°, and ϕ = 0°, though with consideration of error this would likely be repulsive at all values of *R*.

We look to the previously discussed focus on the binding energy of the NO + N_2_ system in both theoretical and experimental studies (Section 1) as a means to compare the efficacy of our PES.^23–25,67,68^ The reported binding energies calculated by Guardado et al. and Lozeille et al. of 335.02 cm^–1^ (EOM-EA-CCSD/d-aug-cc-pVTZ) and 231 cm^–1^ (RCCSD­(T)/aug-cc-pVTZ­(Ryd-2)), respectively, were obtained by taking the difference of the complexes’ global minimum energy for the electronic state of interest and the combined energy of the isolated monomers. This method mirrors how we present Δ*E* for the PES of NO­(*A* ^2^Σ^+^) + N_2_(*X* ^1^Σ_*g*_^+^), therefore our global minimum of 239.51 cm^–1^ can be used as an approximate binding energy for comparison.

Our calculated binding energy most closely resembles the 231 cm^–1^ calculated by Lozeille et al., though this is expected given the similarities in methodology. The differences are our use of the F12 variation of RCCSD­(T) and implementing a CP correction, whereas Lozeille et al. used standard RCCSD­(T), they also used a slightly smaller basis set (of the equivalent of a d-aug-cc-pVTZ) and extrapolated to the CBS limit. Both calculated binding energies sit in good agreement with a total difference of 8 cm^–1^, which is most notable for the efficacy of the F12 variant alongside a CP correction to recover the energy lost to BSIE and BSSE, which Lozeielle et al. recover by extrapolating to the CBS limit. Meanwhile, the reported value of 335.02 cm^–1^, by Guardado et al., sees a greater difference to the values us and Lozeille et al. have calculated, though this can largely be attributed to the fact they made no corrections for BSSE.

As for experimental approaches, these have been predominantly performed using resonance enhanced multiphoton ionization (REMPI) spectroscopy on the *A* ← *X* transition. Multiple studies have been carried out looking to improve the signal-to-noise ratio over the years, beginning with Mack et al. reporting a binding energy of 144 cm^–1^ followed by Lozeille et al. and then most recently Holmes-Ross et al. with binding energies of 143.6 ± −0.5 cm^–1^ and 136.7 ± 3.5 cm^–1^, respectively. All values reported are within good agreement of one another, though notably they follow similar methods of derivation, with changes mostly involving improvement of noise reduction. The issue, as highlighted by Lozeille et al., in their joint theoretical and experimental study, is a large margin of disagreement between the theoretically and experimentally derived binding energy. A portion of this discrepancy is likely due to the lack of zero-point corrections made to any of the presented calculated binding energies. Kłos et al. in their study of the NO­(*A* ^2^Σ^+^) + Ar PES note that the narrow nature of the global energy minimum (≈30–35°) would relate to a large zero point correction.^62^ Whereas for the NO­(*A* ^2^Σ^+^) + N_2_(*X* ^1^Σ_*g*_^+^) PES, we see a much broader minimum energy well, nearly twice as wide as that of the NO­(*A* ^2^Σ^+^) + Ar PES, which would indicate a smaller zero point correction. Though regardless of the scale of the zero-point correction, it is unlikely to account for a ≈100 cm–1 difference and this sentiment is seen in the discussion of both Lozeille et al. and Holmes-Ross et al.^23,67^

Both Lozeille et al. and Holmes-Ross et al. concede that they were unable to find a clear reasoning for the large disparity between the experimental and theoretical binding energies of NO­(*A* ^2^Σ^+^) + N_2_(*X* ^1^Σ_*g*_^+^). Further research in both experimental and theoretical approaches, particularly looking at entirely different approaches to those previously mentioned may bridge the gap. With regards to the theory, although the previously mentioned T1 diagnostics would suggest the PES, at least around the region of the global minimum, is not multireference, it may be beneficial to look to alternative methods such as CASSCF or MRCI. Meanwhile, all currently discussed experimental methods derive the binding energy of the complex through the same method, by interpreting the difference in energy between the “origin point” and the last prominent peak of the *A* ← *X* REMPI spectrum. Exploring alternative experimental approaches could therefore provide new results for comparison.

An alternative would perhaps be to attempt to directly simulate the results of a REMPI spectrum for a more direct comparison. This has been previously performed, with success by Kłos et al. looking at NO­(*A*)-Rg simulated and experimentally derived spectra and binding energies.^62^ Though it should be that they also experience unexpected discrepancies, particularly for the NO­(*A*)-Ar spectrum. In this instance, they noted a consistent displacement of ≈25% and, as such, implemented a scaling factor of 1.23 ^62^ resulting in a better qualitative overlap.

The surfaces produced for both the NO­(*A* ^2^Σ^+^) + O_2_(*X* ^3^Σ_*g*_^–^) and NO­(*A* ^2^Σ^+^) + CO_2_(*X* ^1^Σ_*g*_^+^) collision complexes also provide analogous systems for comparison.^27–29^ Given the similarity in the shape of the partner molecule, it is unsurprising that the NO­(*A* ^2^Σ^+^) + N_2_(*X* ^1^Σ_*g*_^+^) PES most generally closely resembles the PES produced for the NO­(*A* ^2^Σ^+^) + O_2_(*X* ^3^Σ_*g*_^–^) complex which both exhibit two main minimum energy wells, at similar orientations. Two minimum energy wells are present at opposing linear orientations, with the global minimum at the nitrogen facing LN end and the shallower well at the oxygen facing LO end, mirroring what is seen in the NO­(*A* ^2^Σ^+^) + N_2_(*X* ^1^Σ_*g*_^+^) complex. However, the global minimum depth is shallow, reaching only −95 cm^–1^ compared to the −239.51 cm^–1^ depth of the NO­(*A* ^2^Σ^+^) + N_2_(*X* ^1^Σ_*g*_^+^) complex, while the secondary well sees a more comparable depth of ≈20 cm^–1^. Additionally, the repulsive T-shaped, TN, orientation is also present, in which O_2_ and N_2_ make up the head of the T for their respective complexes.

The PES calculated for the NO­(*A* ^2^Σ^+^) + CO_2_(*X*) complex deviates more from the PES of either the NO­(*A*) + O_2_ or NO­(*A*) + N_2_ complexes. The result is a larger number of minimum energy wells and overall a less isotropic surface, though, this is somewhat anticipated given the added complexity of the PES resulting from the additional atom. The impact on the isotropy is such that, none of the cuts of the surface produced are isotropic for the angle of rotation of either partner molecule. The global minimum energy is also substantially deeper, reaching a maximum depth of 823.2 cm^–1^. However, similarities are still present to the other two surfaces, the global minimum energy orientation for the NO­(*A* ^2^Σ^+^) + CO_2_(*X*) complex still occurs near the LN orientation, though in this case it is slightly deviated to θ_NO_ = 140°, rather than fully linear. Additionally, the repulsive T-shaped orientation with the nitrogen facing the CO_2_ (or “TN” orientation) present in both the NO­(*A*) + O_2_ and NO­(*A*) + N_2_ complexes also persists in the NO­(*A* ^2^Σ^+^) + CO_2_(*X*) PES.

From the results of calculations on the NO­(*A* ^2^Σ^+^) + O_2_(*X* ^3^Σ_*g*_^–^) complex, it was suggested that the minimum energy orientations for both the ground state and excited states were predominantly due to minimization of electronic repulsion from preferable dipole–quadrupole interactions. This appears consistent for the NO­(*A*) + N_2_ collision complex as the minimum energy orientations and signs of the dipole/quadrupole moments match those of the NO­(*A*) + O_2_. Notably, we also observe a trend between the size of the quadrupole moment and minimum energy well depth also points to dipole–quadrupole interactions predominantly effecting the characteristics of the PES. The quadrupole moment of the partner molecules becomes more negative along the pattern of O_2_, N_2_, and finally CO_2_ (ca. −1.1, −5.0 and −14.1 × 10^–40^ cm^2^, respectively).^28,29^ In turn, the well depth of the global minimum for their respective NO­(*A*) collision complexes increases along the same pattern (−95 cm^–1^, −239.51 cm^–1^, and ≈−830 cm^–1^, respectively).

We now discuss the PES in the context of recent experimental work on the NO­(*A* ^2^Σ^+^) + N_2_(*X* ^1^Σ_*g*_^+^) complex. Two separate groups have recently reported experiments on the photodissociation of NO­(*A* ^2^Σ^+^) + N_2_(*X* ^1^Σ_*g*_^+^) VdW complexes, VMI of NO­(*A* ^2^Σ^+^) products to resolve the kinetic energy distribution and translational anisotropy at a range of excess energies.^26,67,69,70^ The NO­(*X* ^2^Π) + N_2_(*X* ^1^Σ_*g*_^+^) complex is known, from IR spectroscopy and the calculated RCCSD­(T) PES, to adopt a T-shaped geometry, with the NO forming the top bar of the T, with the potential minimum at *R* = 3.9 Å.^68^ This is the geometry to which we refer in this paper as the “Hammer.” Figure 3 shows that the NO­(*A* ^2^Σ^+^) + N_2_(*X* ^1^Σ_*g*_^+^) PES in the H-geometry is repulsive along all *R*, providing an initial outward trajectory on the PES. However, the PES also has strong anisotropy with respect to NO rotation at this *R*, as shown in the “LN to H to LO” surface in Figure 3, where the LN minimum is easily accessible. We would therefore expect substantial rotational excitation of the NO­(*A* ^2^Σ^+^) in the dissociation. This is consistent with the reported experimental NO­(*A* ^2^Σ^+^) rotational excitation, which was observed to extend up to the energetic limit for available energies ranging from 28 cm^–1^ to 758 cm^–1^.^70^ Holmes-Ross et al. reported correlated NO–N_2_ rotational state distributions for excess energies of 90 and 190 cm^–1^.^67^ Significant N_2_ rotational excitation was observed, including anticorrelation for low-*J* NO where the coincident N_2_ distribution peaked at high-*J*. This is perhaps surprising, as the PES presented here is less anisotropic with respect to N_2_ rotation when starting from the Hammer geometry, as shown in the “H to P to H” and “H to C to H” surfaces in Figure 4. Overall, we would therefore expect that less of the available energy will be transferred to N_2_ rotation. However, as discussed by Holmes-Ross et al., in these qualitative arguments we have ignored the large amplitude motion of the ‘floppy’ NO­(*X* ^2^Π) + N_2_(*X* ^1^Σ_*g*_^+^) complex and the associated Franck–Condon overlap with a wide range of initial NO­(*A* ^2^Σ^+^) + N_2_(*X* ^1^Σ_*g*_^+^) geometries. The high-quality NO­(*A* ^2^Σ^+^) + N_2_(*X* ^1^Σ_*g*_^+^) PES presented here, combined with the literature NO­(X) + N_2_(*X* ^1^Σ_*g*_^+^) PES, provide the electronic structure information required for classical or quantum calculations of the photodissociation dynamics. We hope that this will stimulate further experimental and theoretical studies of the dynamics of the NO–N_2_ complex.

A significant motivation for the calculations presented here was our recent experimental study of NO­(*A* ^2^Σ^+^) RET in collisions with N_2_(*X* ^1^Σ_*g*_^+^).^21,22^ These experiments combined crossed molecular beam scattering with velocity-map ion-imaging, to determine differential scattering cross sections (DCSs) for NO­((*A* ^2^Σ^+^), *v* = 0, *N* = 0) to a range of specified final NO­((*A* ^2^Σ^+^), *N*′) rotational states, *N*′ = 3 and 5 to 11. For each final state, the DCS was determined as a function of the internal energy transferred to the unobserved N_2_, through the analysis of the velocity-map images constrained by energy and linear momentum conservation. The images and resulting DCSs showed a very sharp forward-scattered peak for *N*′ ≤ 10, correlated with the low rotational excitation of the N_2_. This forward scattering with NO­(*A* ^2^Σ^+^) rotational excitation was also observed in our earlier experiments on NO­(*A* ^2^Σ^+^) + Ar and NO­(*A* ^2^Σ^+^) + Kr scattering, where quantum scattering calculations on accurate ab initio PESs show it can be assigned to glory-type scattering from an attractive well centered around linear geometries, particular at the N-end of NO­(*A* ^2^Σ^+^).^18,71^ We therefore suggest that the forward-scattered peak observed in NO­(*A* ^2^Σ^+^) + N_2_(*X* ^1^Σ_*g*_^+^) scattering is similarly generated by long-range glory-scattering collisions that sample the attractive well in the LN geometry. We have also seen strong forward scattering in NO­(*A* ^2^Σ^+^) + CO_2_(*X* ^1^Σ_*g*_^+^) collisions, where in contrast both collision partners undergo significant correlated rotational excitation.^72^ We attributed this to the deep attractive well of the NO­(*A* ^2^Σ^+^) + CO_2_(*X* ^1^Σ_*g*_^+^) PES, which has strong anisotropy with respect to rotation of both NO and CO_2_.^28^ It is therefore somewhat of a surprise that N_2_ is not significantly rotationally excited in these long-range collisions, given the strong anisotropy seen in the “LN to TN to LN” cut.

Scattering into high-*N*′ NO final states was correlated with increased N_2_ rotational excitation, and a broader range of scattering angles consistent with repulsive-wall rainbow scattering. However, even the highest NO rotational states were not correlated with equivalent levels of N_2_ rotational excitation, even though no significant constraint was applied by the available energy. This is consistent with the general observation that the PES has a lower anisotropy, particularly in the repulsive wall, with respect to rotation of N_2_ than it does with respect to NO, and hence that we would expect overall lower rotational excitation of N_2_ in NO­(*A* ^2^Σ^+^) + N_2_(*X* ^1^Σ_*g*_^+^) collisions. Scattering calculations, either classical or quantum, using this new ab initio PES would provide quantitative arguments to support, or refute, these qualitative interpretations, and we hope that the publication of this PES will stimulate such calculations.

## 4 Conclusion

An extensive exploration of the VdW region of the NO­(*A* ^2^Σ^+^) + N_2_(*X* ^1^Σ_*g*_^+^) collision complex has been carried out using CCSD­(T)-F12/t-aug-cc-pVTZ with consideration of BSSE effects through the use of an explicitly correlated method alongside CP corrections. A broad minimum energy well is observed along the linear nitrogen-to-nitrogen facing, or LN, orientation, spanning a rotation of θ_NN_ ≈ 90° and *R* ≈ 3 Å. The global minimum was found to reach a maximum depth of Δ*E* = −239.51 cm^–1^ at R=4.2⁡Å , θ_NO_ = 180°, θ_NN_ = 0°, and ϕ = 0° surrounding the linear nitrogen-to-nitrogen (LN) facing orientation. Ultimately, the newly calculated PES will benefit further scattering calculations, and in particular the interpretation of recent crossed molecular beam studies on this system.

## Supplementary Material



## Notes

Benchmarking results for the method and basis set testing, T1 diagnostic breakdown for the produced PES, additional cuts of the PES at intermediary angles to those presented in the main publication, contour plots for all cuts of PES, and data calculated for the PES (PDF)
