# Supplementary material for: Exploration of the van der Waals Region of the NO A 2Σ+ + N2 X 1Σ g + Collision Complex
Source: J Phys Chem A. 2026 Jun 29;130(28):5334–45. doi: 10.1021/acs.jpca.6c01405 (PMC13383751; doi:10.1021/acs.jpca.6c01405)
Supplement: Supplementary file 1 [file jp6c01405_si_001.pdf]

Supporting Information for Publication: Exploration of the van der Waals region of the NO$A^2\Sigma^+$ + $N_2X^1\Sigma_g^+$ collision complex

Alexandre De Matos Loja, Matthew L. Costen, Martin J. Paterson.*
Institute of Chemical Sciences, Heriot-Watt University, Edinburgh, EH14 4AS, UK.
Email:

1 Method and basis testing

The full set of results for the benchmarking mentioned in the main text is provided below in Table S1.

	Non-BSSE-corrected		BSSE-corrected	
Method	R_{vdW} ($\AA$)	ΔE(cm^{-1})	R_{vdW} ($\AA$)	ΔE(cm^{-1})
CCSD(T)/aug-cc-pVTZ	4.2	1246.5	4.3	181.2
CCSD(T)/d-aug-cc-pVTZ	4.2	430.3	4.3	229.2
CCSD(T)/t-aug-cc-pVTZ	4.2	424.6	4.3	231.8
PNO-CCSD(T)/aug-cc-pVTZ	4.3	1224.6	4.4	186.4
PNO-CCSD(T)/d-aug-cc-pVTZ	4.3	383.5	4.3	226.9
PNO-CCSD(T)/t-aug-cc-pVTZ	4.3	373.2	4.3	235.6
CCSD(T)-F12/aug-cc-pVTZ	4.2	764.3	4.3	155.1
CCSD(T)-F12/d-aug-cc-pVTZ	4.2	272.4	4.2	238.8
CCSD(T)-F12/t-aug-cc-pVTZ	4.2	271.5	4.2	239.4

Table S1: Complete comparison of vdW minima for scans of the linear-nitrogen (LN) orientation of $NO(A^2\Sigma^+) + N_2(X^1\Sigma^+)$ along chosen R values, calculated for benchmarking method and basis set used in producing the full PES. "BSSE-corrected" results include a counter-poise correction to the final energy using the Boys-Bernardi method[1].

2 T1 diagnostics

As mentioned in the main text, alongside all points calculated, the T1 diagnostic was calculated to approximate the involvement of multi-reference character and thus the efficiency of coupled cluster to accurately describe the wavefunction. Due to a minor loss of data, only $23,188$ T1 values were collected of the total $23,777$ points calculated. Of the T1 values calculated a maximum value of 0.0276 was observed and a minimum of 0.0192 with an average value across all points of 0.0215. As mentioned in the main text, the majority of the surface sits above the 0.02 threshold, with $\approx 85\%$ of the surface having a T1 value above 0.02. However, for the location on the PES surrounding the main minimum

energy well along the cut at $\theta_{NO} = 180°$, $\theta_{NN} = 0°$ and $\phi = 0°$, the T1 value of the points are firmly below 0.02 from R values below $8\mathring{A}$. The exact minimum energy point itself sees a low T1 value of 0.0194 which again is firmly below the 0.02 threshold so the efficacy of our method to calculate this point is not in question. A more detailed breakdown of the T1 diagnostics of the surface is provided in the table below.

	R range			
	$15\mathring{A} \rightarrow 4.2\mathring{A}$		$4.1\mathring{A} \rightarrow 3.2\mathring{A}$	
T1 threshold	N^{o} points	% of range	N^{o} points	% of range
total	15708	68 (of total)	7480	32 (of total)
<0.02	2733	17	698	9
<0.021	9925	63	1151	15
<0.022	12000	76	1689	23

3 Additional PES cuts

Additional cuts of the PES were calculated, filling out additional possible orientations outside of the ones highlighted in the main text. Here all surfaces are presented alongside one another, including those already presented in the main text. Cuts of the PES were also calculated for orientations outside of those outlined in the main text. Here we present all surfaces provided together, along with the additional surfaces not shown in the main text.

The majority of these surfaces have been produced with a larger angular step size ranging from $5° \rightarrow 20°$. As with the surfaces presented in the main text, 31 values of R are used within the range of $15\mathring{A} \rightarrow 3.2\mathring{A}$ at varying levels of separation including the values [15, 13, 11, 9, 8, 7, 6.5, 6, 5.8, 5.6, 5.4, 5.2, 5, 4.9, 4.8, 4.7, 4.6, 4.5, 4.4, 4.3, 4.2, 4.1, 4, 3.9, 3.8, 3.7, 3.6, 3.5, 3.4, 3.3, 3.2].

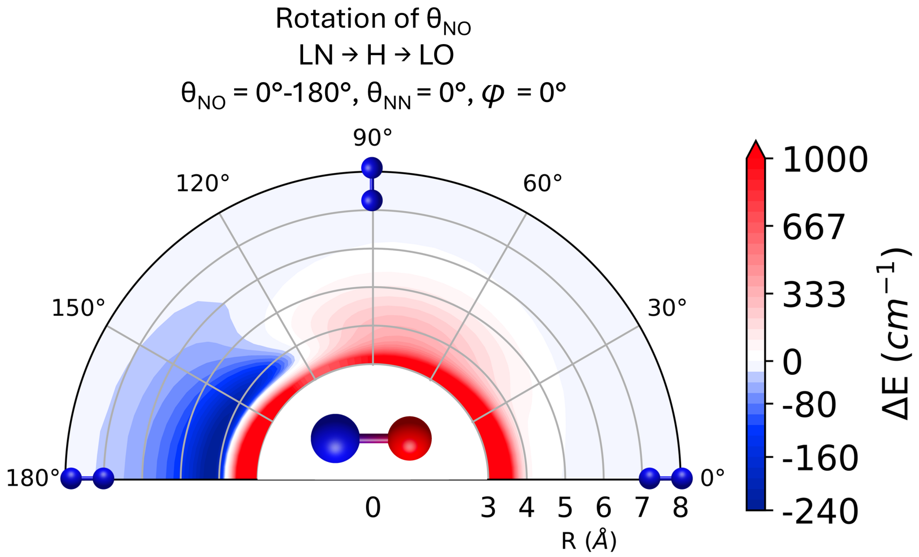

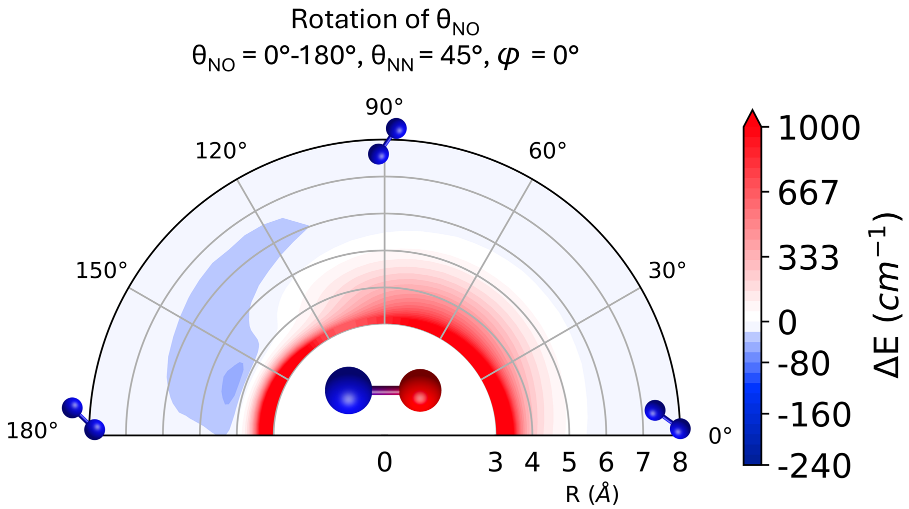

Figure S1: Three-dimensional cuts of the calculated PES for the $NO(A^2\Sigma^+) + N_2(X^1\Sigma^+)$ for rotation of the Θ_{NO} angle. Fixed angles are given at the footer of each plot. Further plot details are elaborated in the main text.

Figure S2: Three-dimensional cuts of the calculated PES for the $NO(A^2\Sigma^+) + N_2(X^1\Sigma^+)$ for rotation of the Θ_{NO} angle. Fixed angles are given at the footer of each plot. Further plot details are elaborated in the main text.

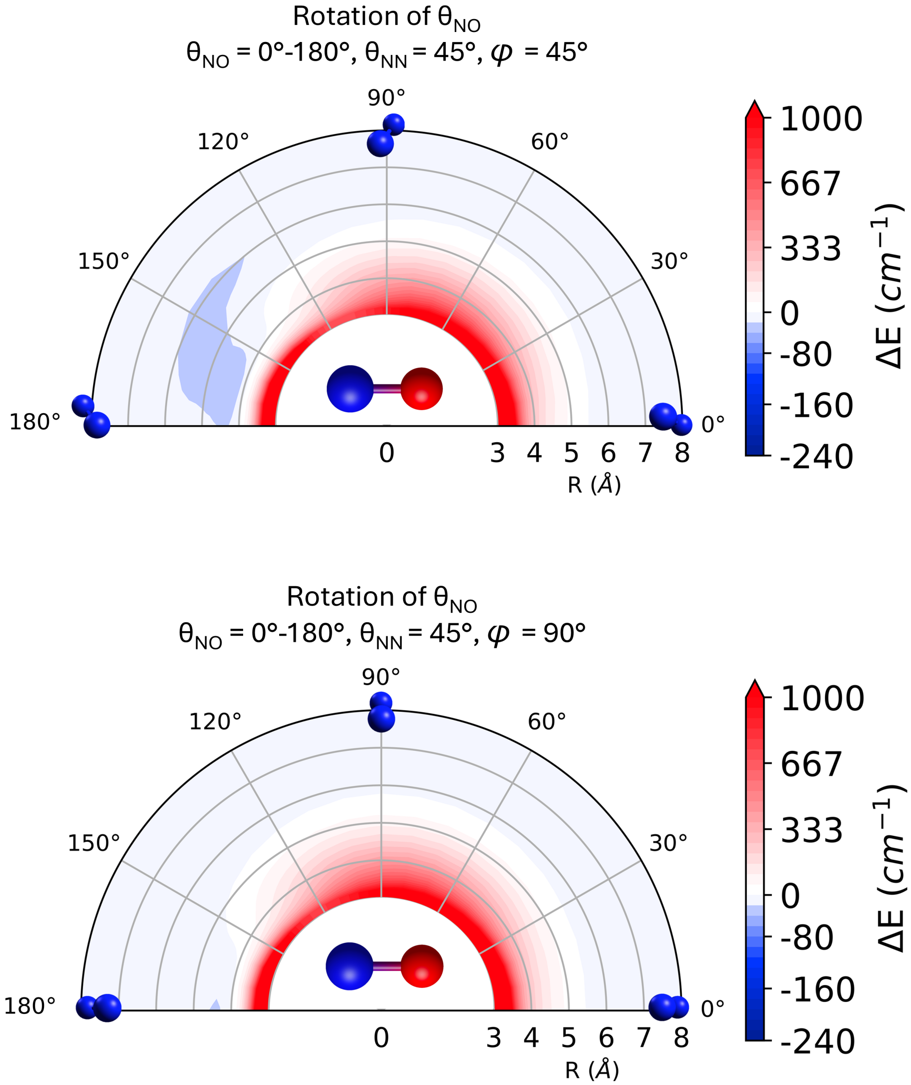

Figure S3: Three-dimensional cuts of the calculated PES for the $NO(A^2\Sigma^+) + N_2(X^1\Sigma^+)$ for rotation of the Θ_{NO} angle. Fixed angles are given at the footer of each plot. Further plot details are elaborated in the main text.

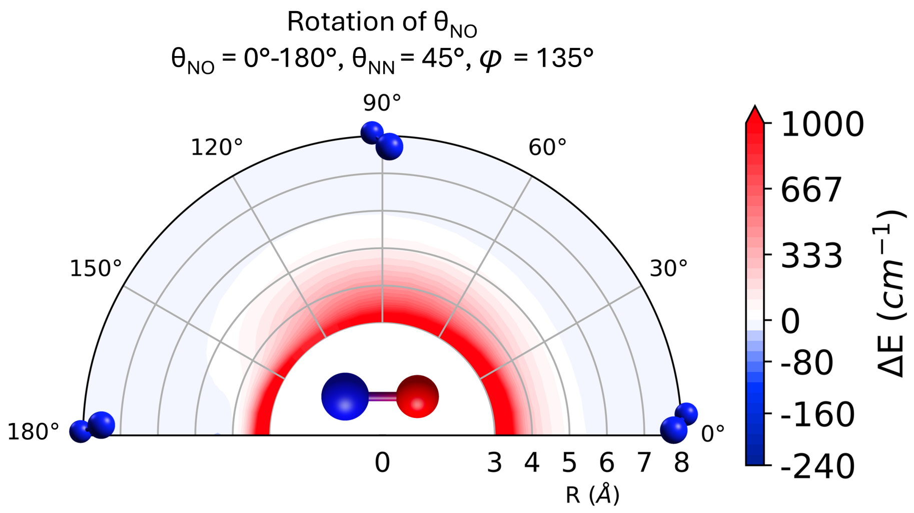

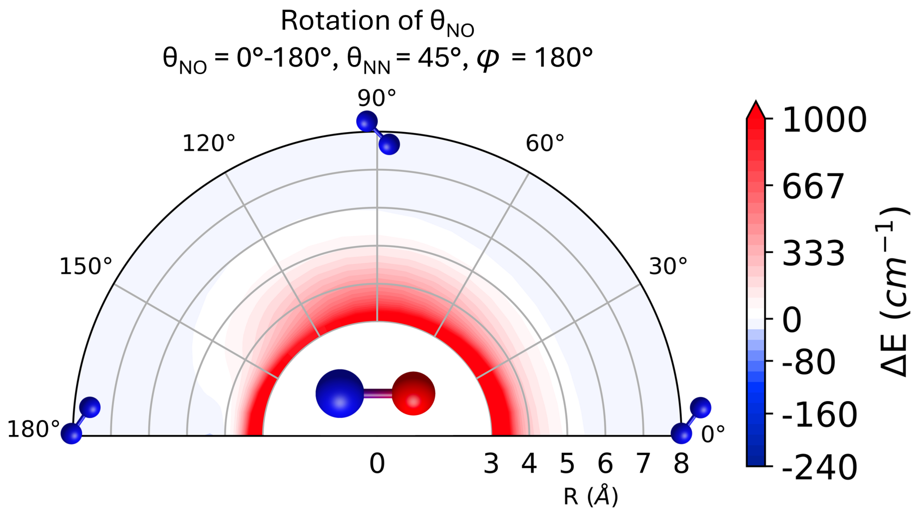

Figure S4: Three-dimensional cuts of the calculated PES for the $NO(A^2\Sigma^+) + N_2(X^1\Sigma^+)$ for rotation of the Θ_{NO} angle. Fixed angles are given at the footer of each plot. Further plot details are elaborated in the main text.

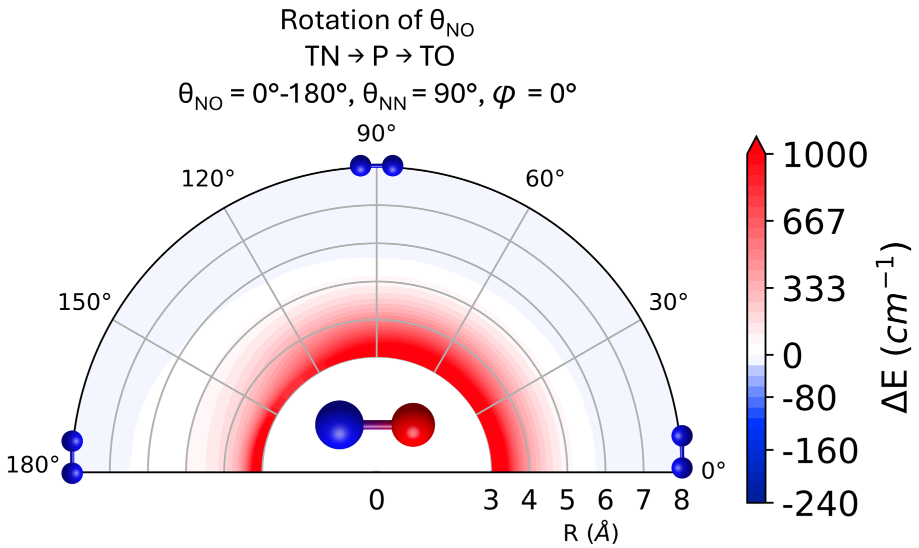

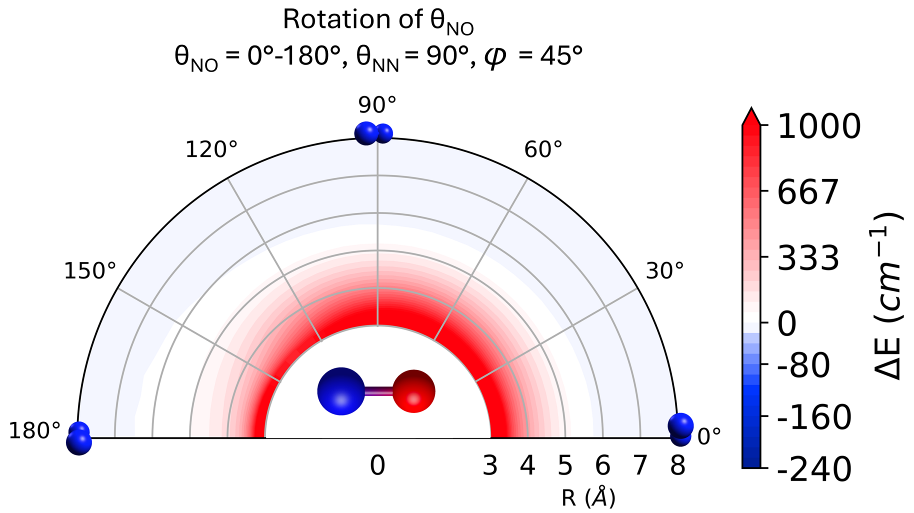

Figure S5: Three-dimensional cuts of the calculated PES for the $NO(A^2\Sigma^+) + N_2(X^1\Sigma^+)$ for rotation of the Θ_{NO} angle. Fixed angles are given at the footer of each plot. Further plot details are elaborated in the main text.

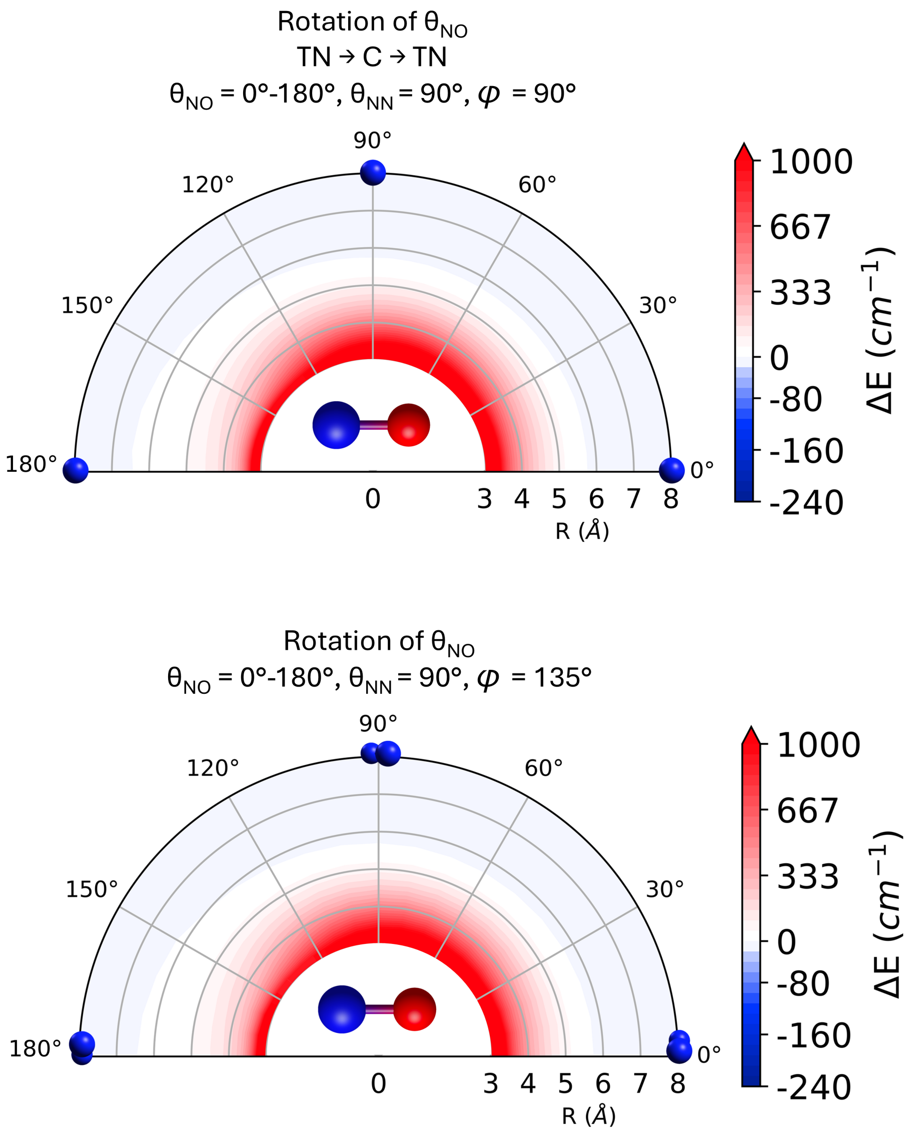

Figure S6: Three-dimensional cuts of the calculated PES for the $NO(A^2\Sigma^+) + N_2(X^1\Sigma^+)$ for rotation of the Θ_{NO} angle. Fixed angles are given at the footer of each plot. Further plot details are elaborated in the main text.

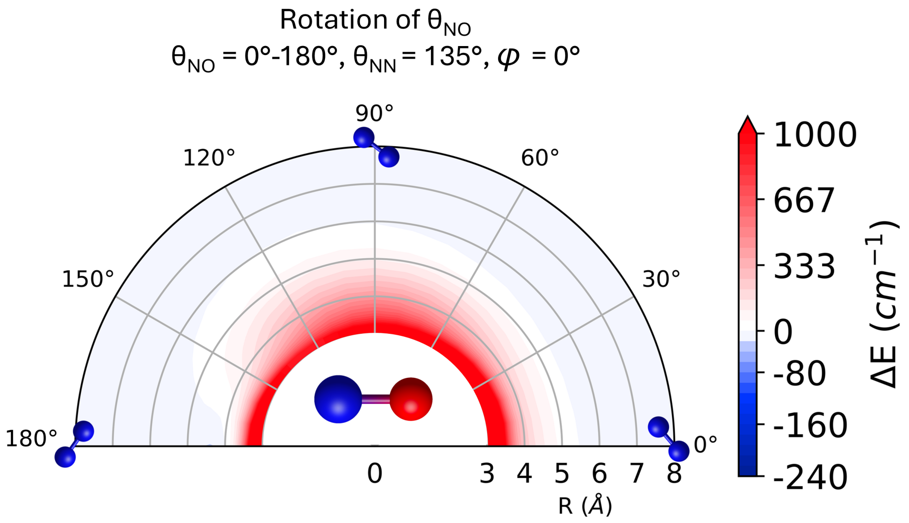

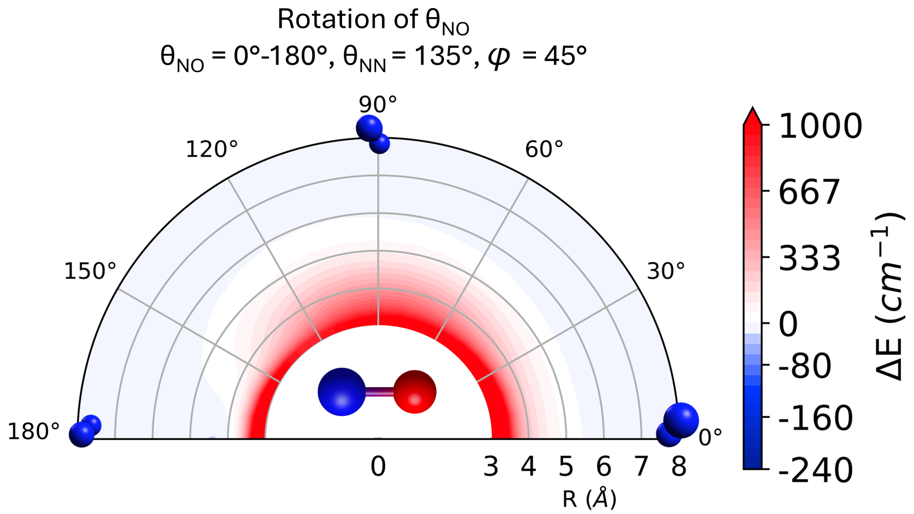

Figure S7: Three-dimensional cuts of the calculated PES for the $NO(A^2\Sigma^+) + N_2(X^1\Sigma^+)$ for rotation of the Θ_{NO} angle. Fixed angles are given at the footer of each plot. Further plot details are elaborated in the main text.

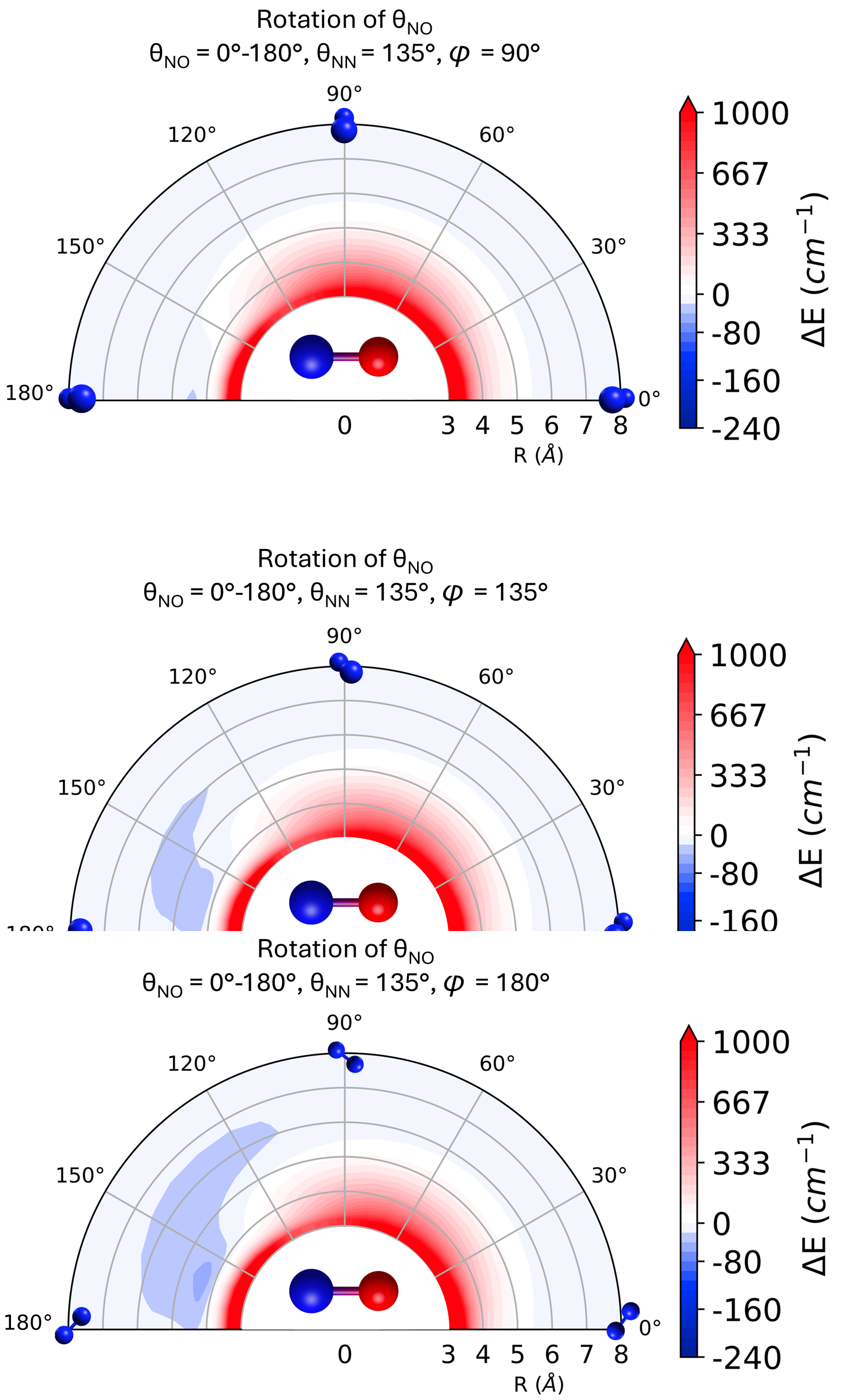

Figure S9: Three-dimensional cut of the calculated PES for the $NO(A^2\Sigma^+) + N_2(X^1\Sigma^+)$ for rotation of the Θ_{NO} angle. Fixed angles are given at the footer of each plot. Further plot details are elaborated in the main text.

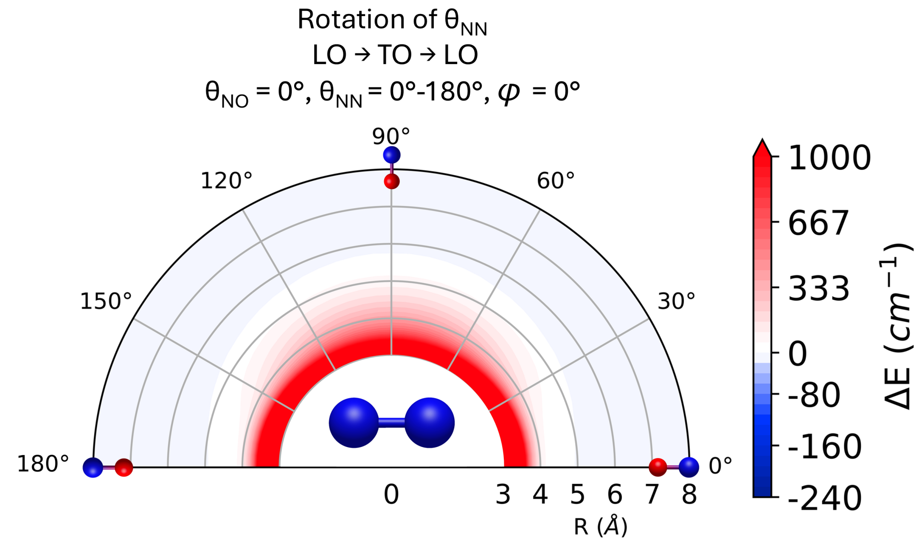

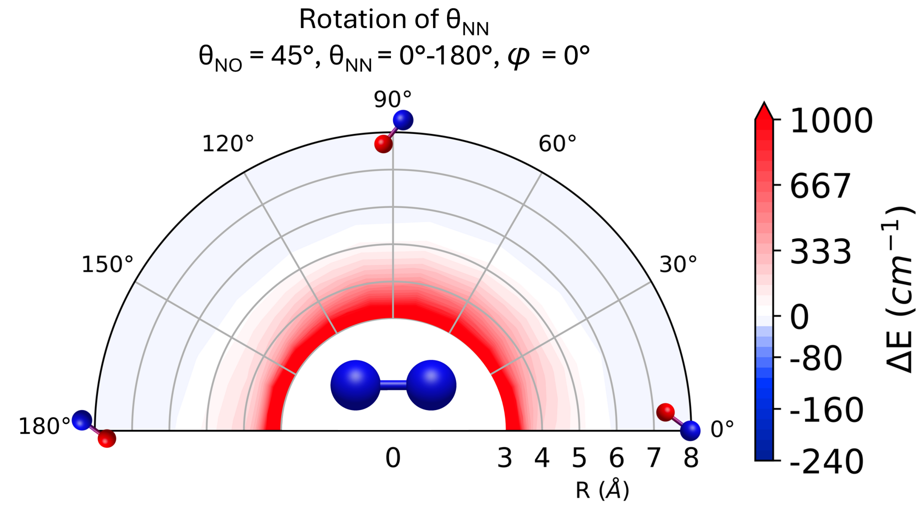

Figure S10: Three-dimensional cuts of the calculated PES for the $NO(A^2\Sigma^+) + N_2(X^1\Sigma^+)$ for rotation of the Θ_{NN} angle. Fixed angles are given at the footer of each plot. Further plot details are elaborated in the main text.

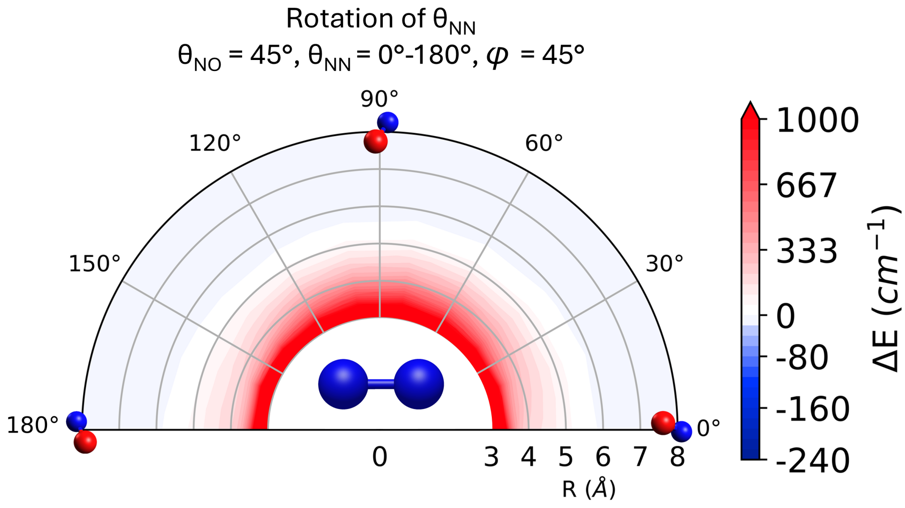

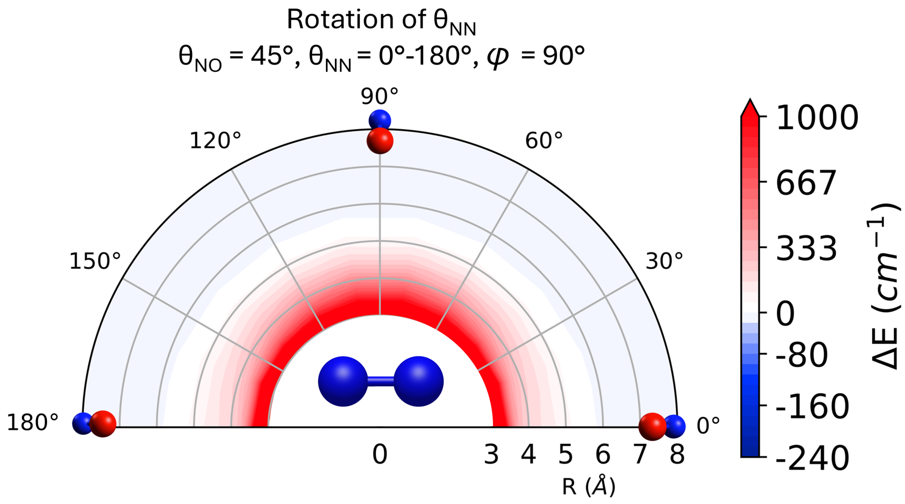

Figure S11: Three-dimensional cuts of the calculated PES for the $NO(A^2\Sigma^+) + N_2(X^1\Sigma^+)$ for rotation of the Θ_{NN} angle. Fixed angles are given at the footer of each plot. Further plot details are elaborated in the main text.

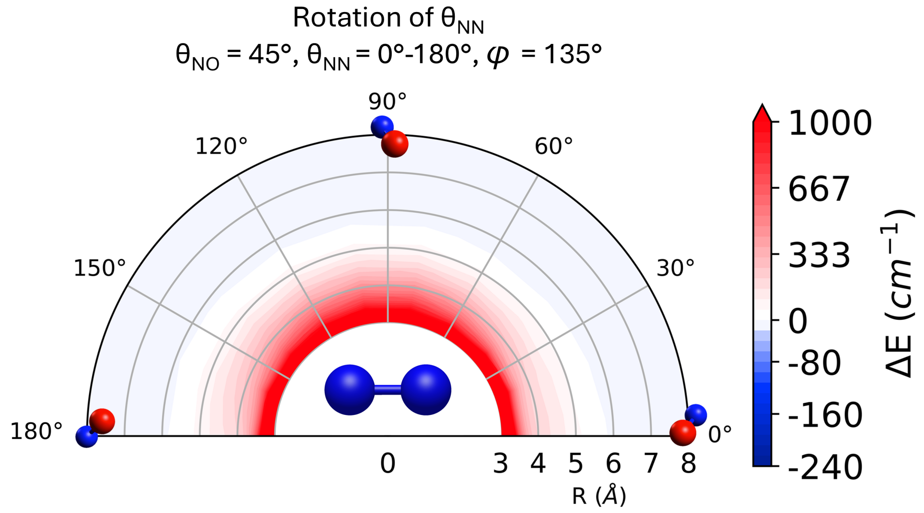

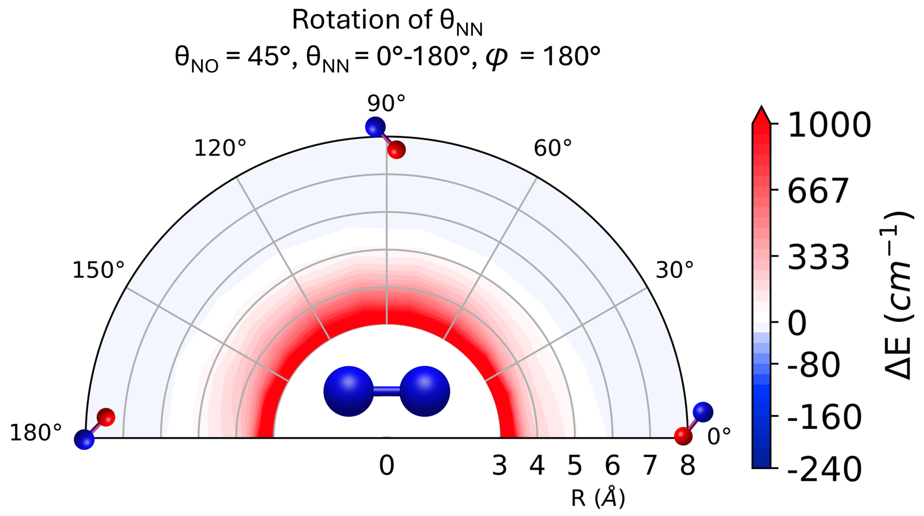

Figure S12: Three-dimensional cuts of the calculated PES for the $NO(A^2\Sigma^+) + N_2(X^1\Sigma^+)$ for rotation of the Θ_{NN} angle. Fixed angles are given at the footer of each plot. Further plot details are elaborated in the main text.

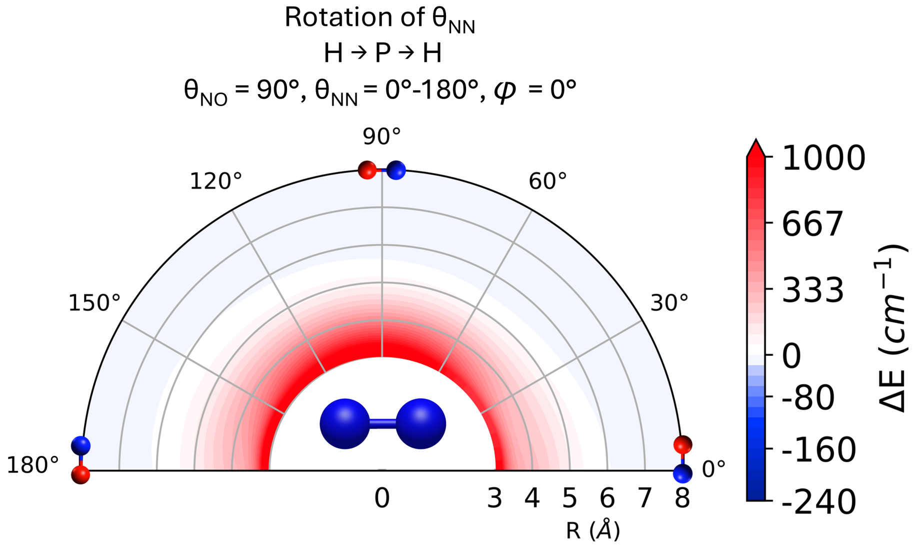

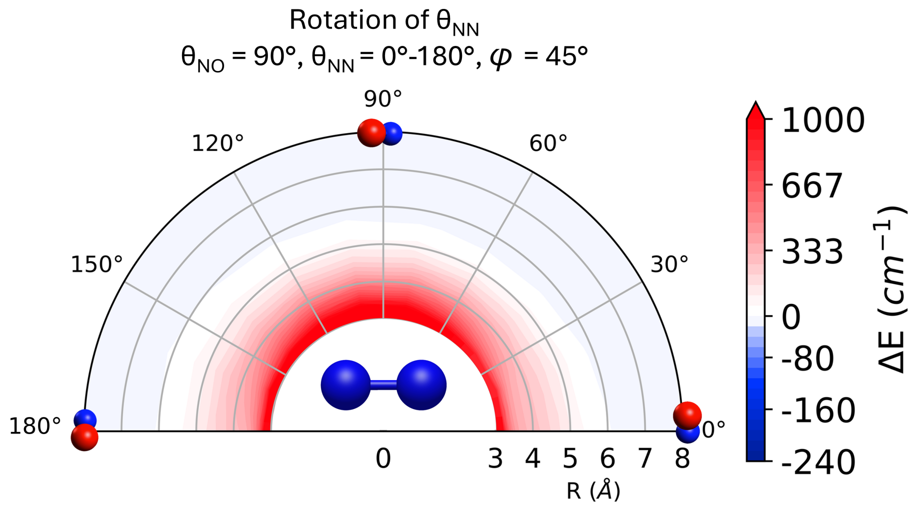

Figure S13: Three-dimensional cuts of the calculated PES for the $NO(A^2\Sigma^+) + N_2(X^1\Sigma^+)$ for rotation of the Θ_{NN} angle. Fixed angles are given at the footer of each plot. Further plot details are elaborated in the main text.

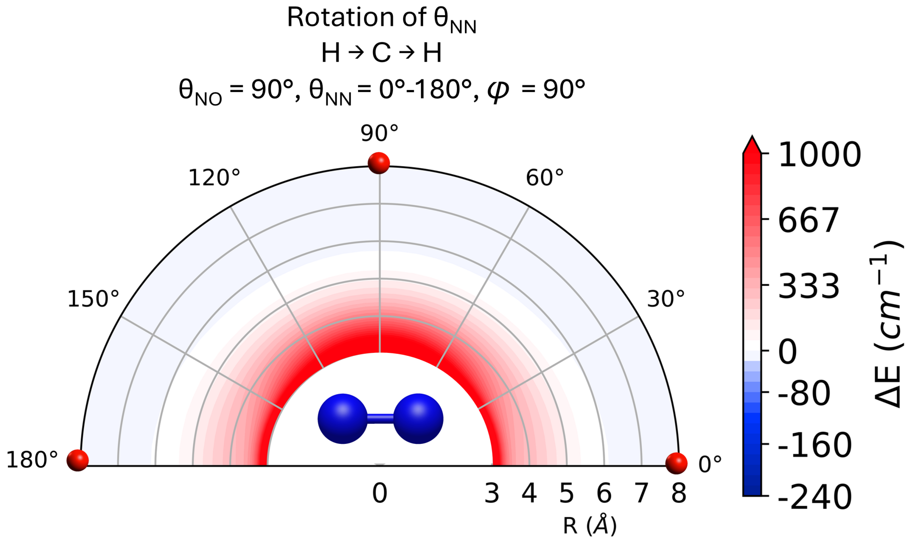

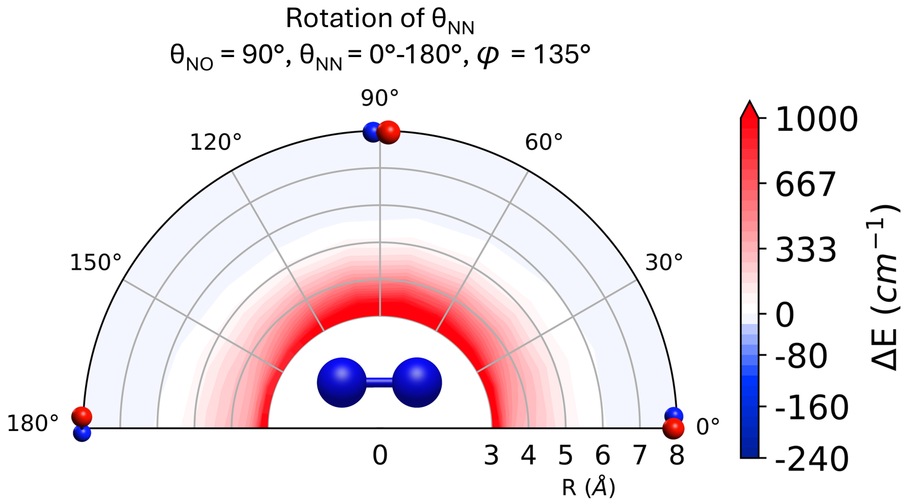

Figure S14: Three-dimensional cuts of the calculated PES for the $NO(A^2\Sigma^+) + N_2(X^1\Sigma^+)$ for rotation of the Θ_{NN} angle. Fixed angles are given at the footer of each plot. Further plot details are elaborated in the main text.

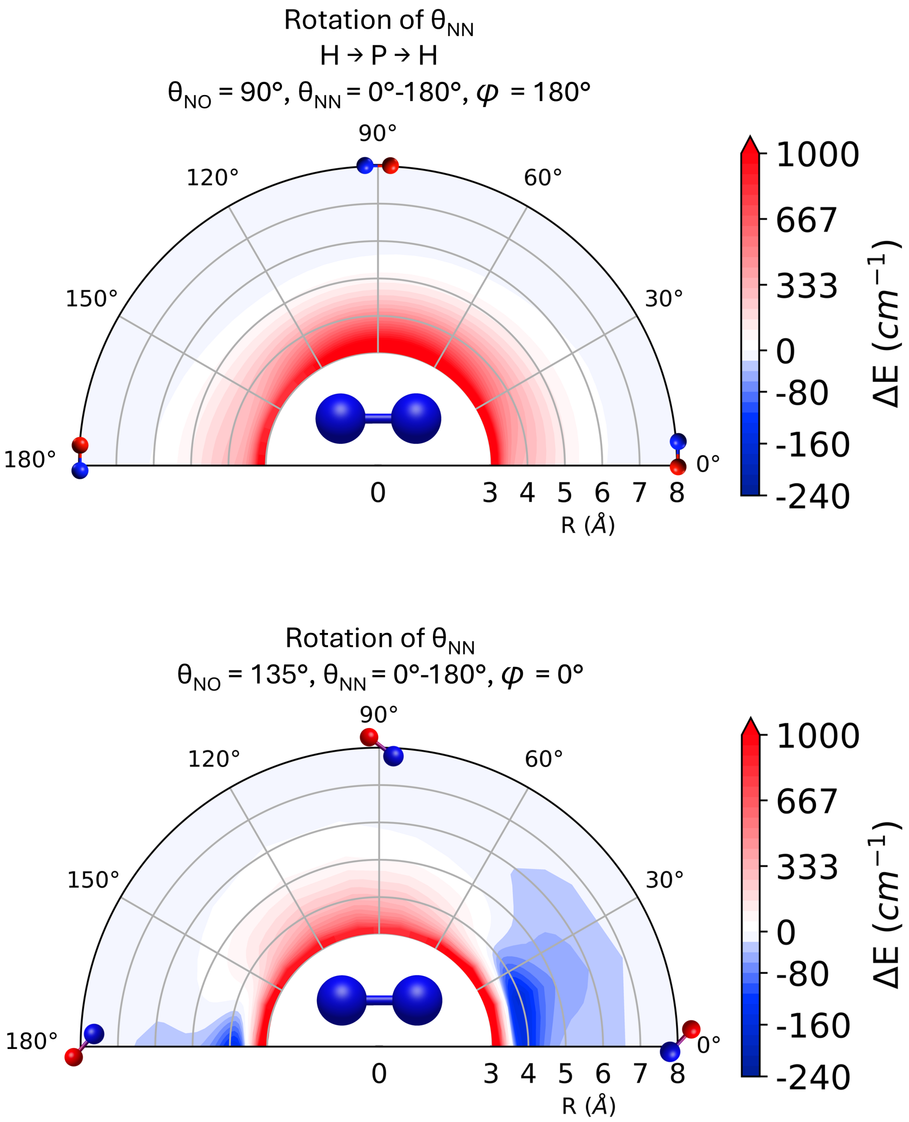

Figure S15: Three-dimensional cuts of the calculated PES for the $NO(A^2\Sigma^+) + N_2(X^1\Sigma^+)$ for rotation of the Θ_{NN} angle. Fixed angles are given at the footer of each plot. Further plot details are elaborated in the main text.

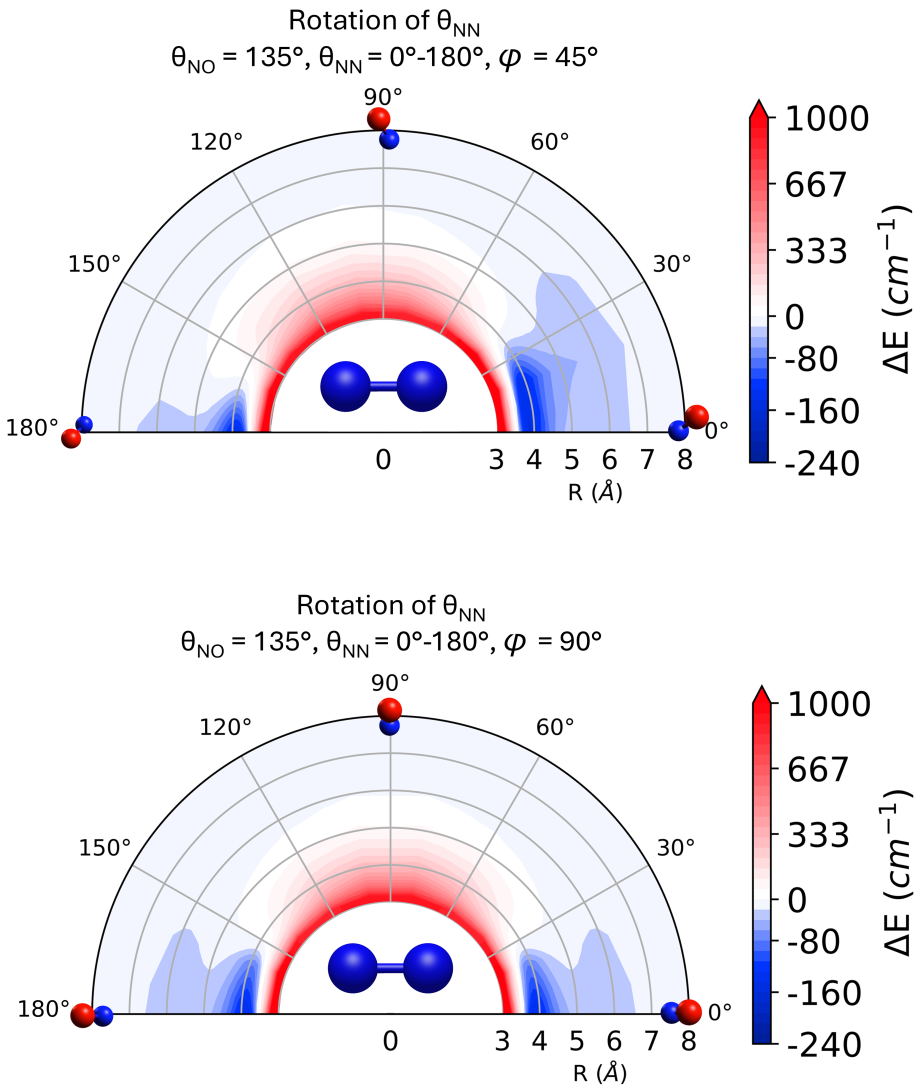

Figure S16: Three-dimensional cuts of the calculated PES for the $NO(A^2\Sigma^+) + N_2(X^1\Sigma^+)$ for rotation of the Θ_{NN} angle. Fixed angles are given at the footer of each plot. Further plot details are elaborated in the main text.

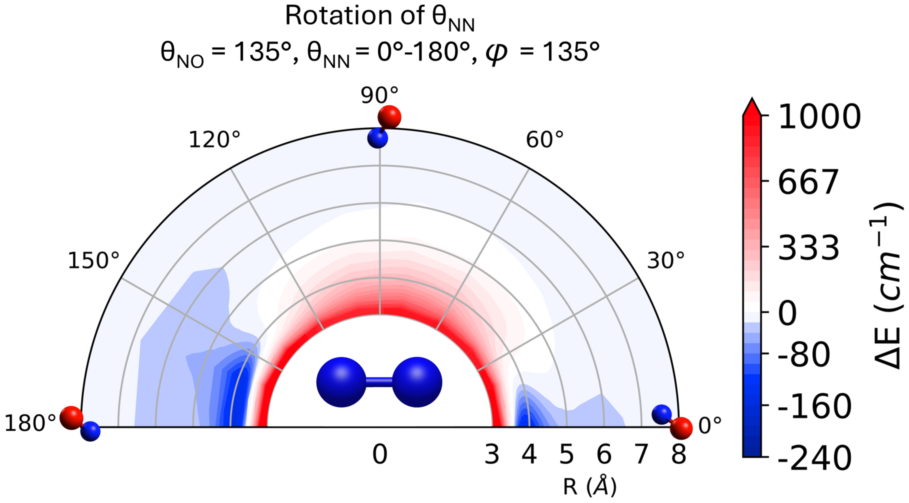

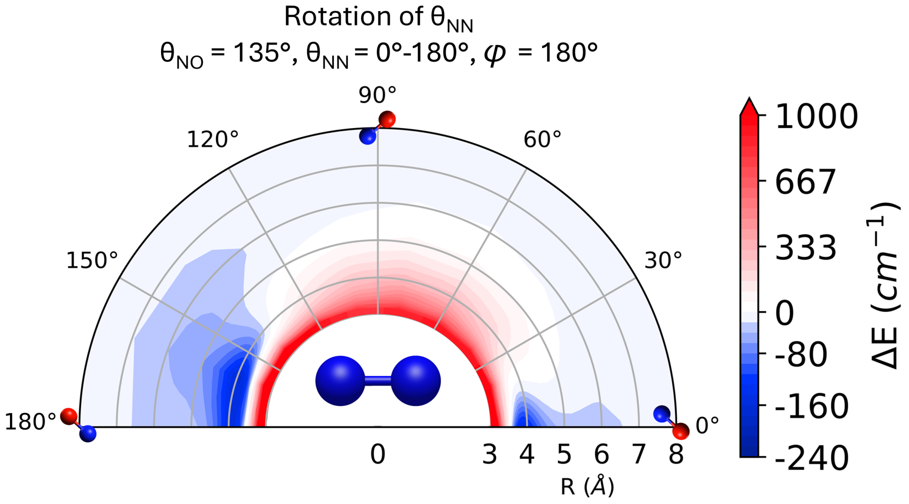

Figure S17: Three-dimensional cuts of the calculated PES for the $NO(A^2\Sigma^+) + N_2(X^1\Sigma^+)$ for rotation of the Θ_{NN} angle. Fixed angles are given at the footer of each plot. Further plot details are elaborated in the main text.

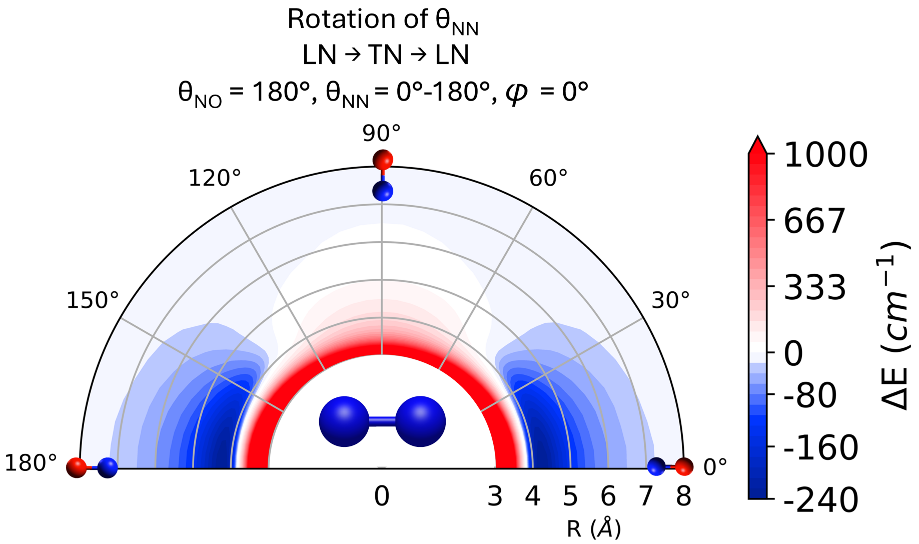

Figure S18: Three-dimensional cut of the calculated PES for the $NO(A^2\Sigma^+) + N_2(X^1\Sigma^+)$ for rotation of the Θ_{NN} angle. Fixed angles are given at the footer of each plot. Further plot details are elaborated in the main text.

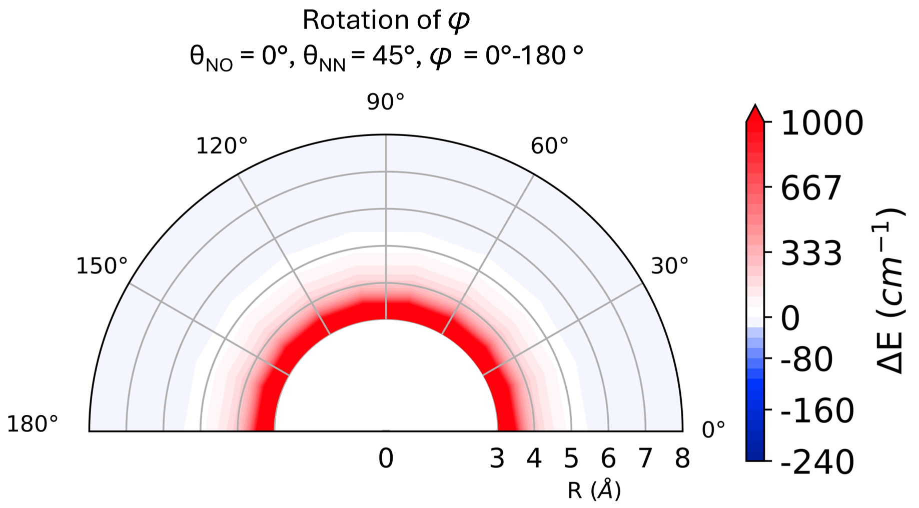

Figure S19: Three-dimensional cut of the calculated PES for the $NO(A^2\Sigma^+) + N_2(X^1\Sigma^+)$ for rotation of the ψ angle. Fixed angles are given at the footer of each plot. Further plot details are elaborated in the main text.

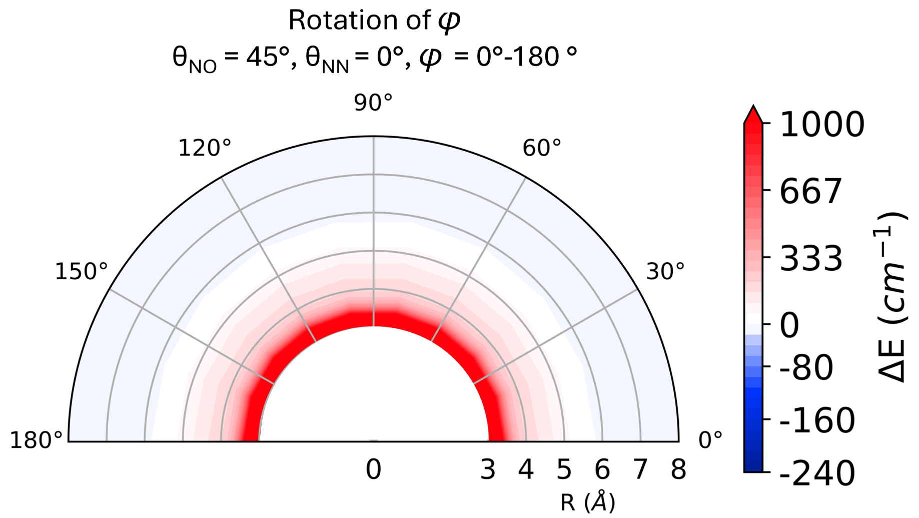

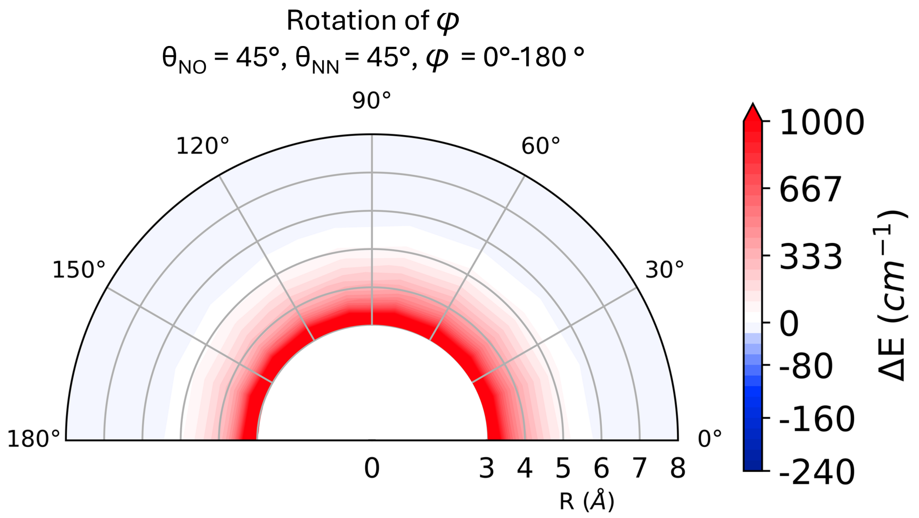

Figure S20: Three-dimensional cuts of the calculated PES for the $NO(A^2\Sigma^+) + N_2(X^1\Sigma^+)$ for rotation of the ψ angle. Fixed angles are given at the footer of each plot. Further plot details are elaborated in the main text.

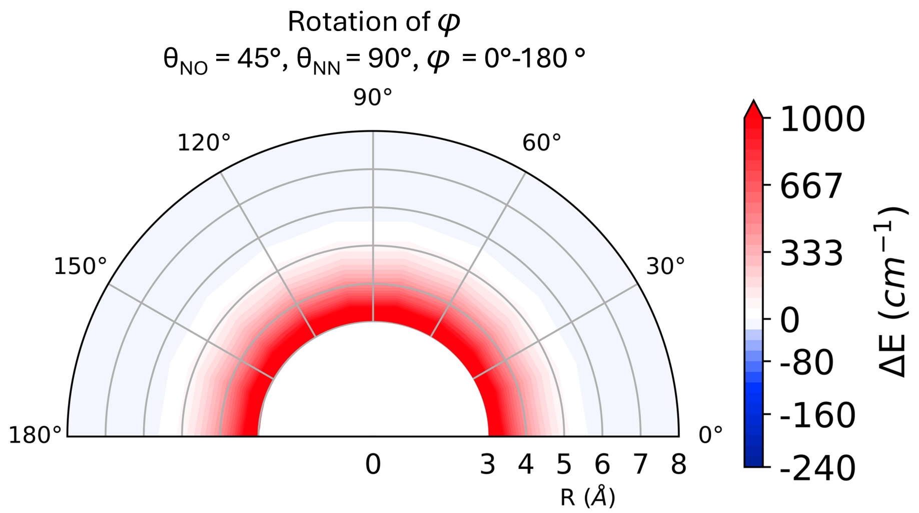

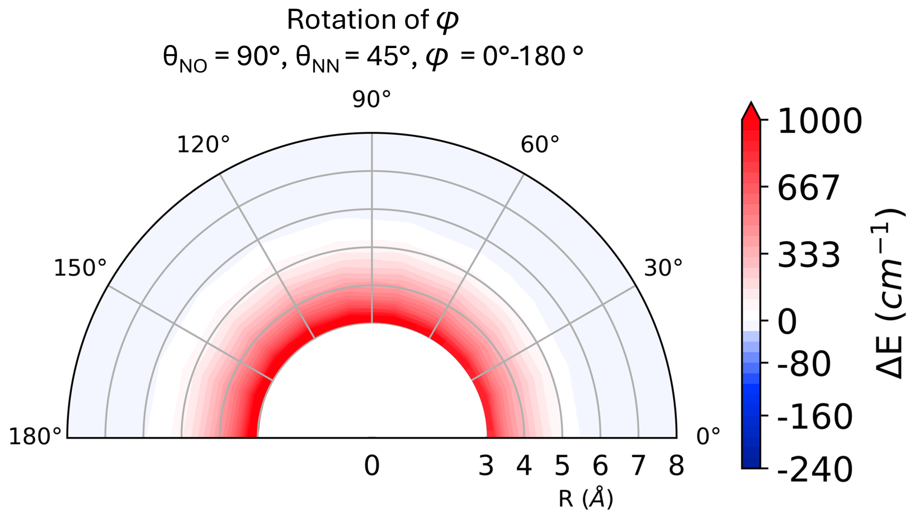

Figure S21: Three-dimensional cuts of the calculated PES for the $NO(A^2\Sigma^+) + N_2(X^1\Sigma^+)$ for rotation of the ψ angle. Fixed angles are given at the footer of each plot. Further plot details are elaborated in the main text.

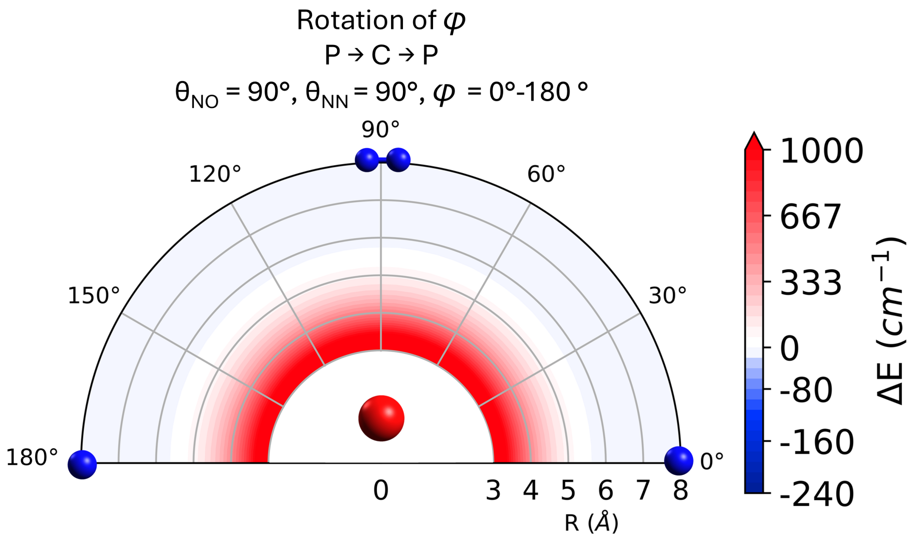

Figure S22: Three-dimensional cuts of the calculated PES for the $NO(A^2\Sigma^+) + N_2(X^1\Sigma^+)$ for rotation of the ψ angle. Fixed angles are given at the footer of each plot. Further plot details are elaborated in the main text.

4 Contour Plots

The previously presented PES cuts in the main text (Figs. [3]-[5]) and in the SI (Figs. S1-S22) are presented here also as contour plots.

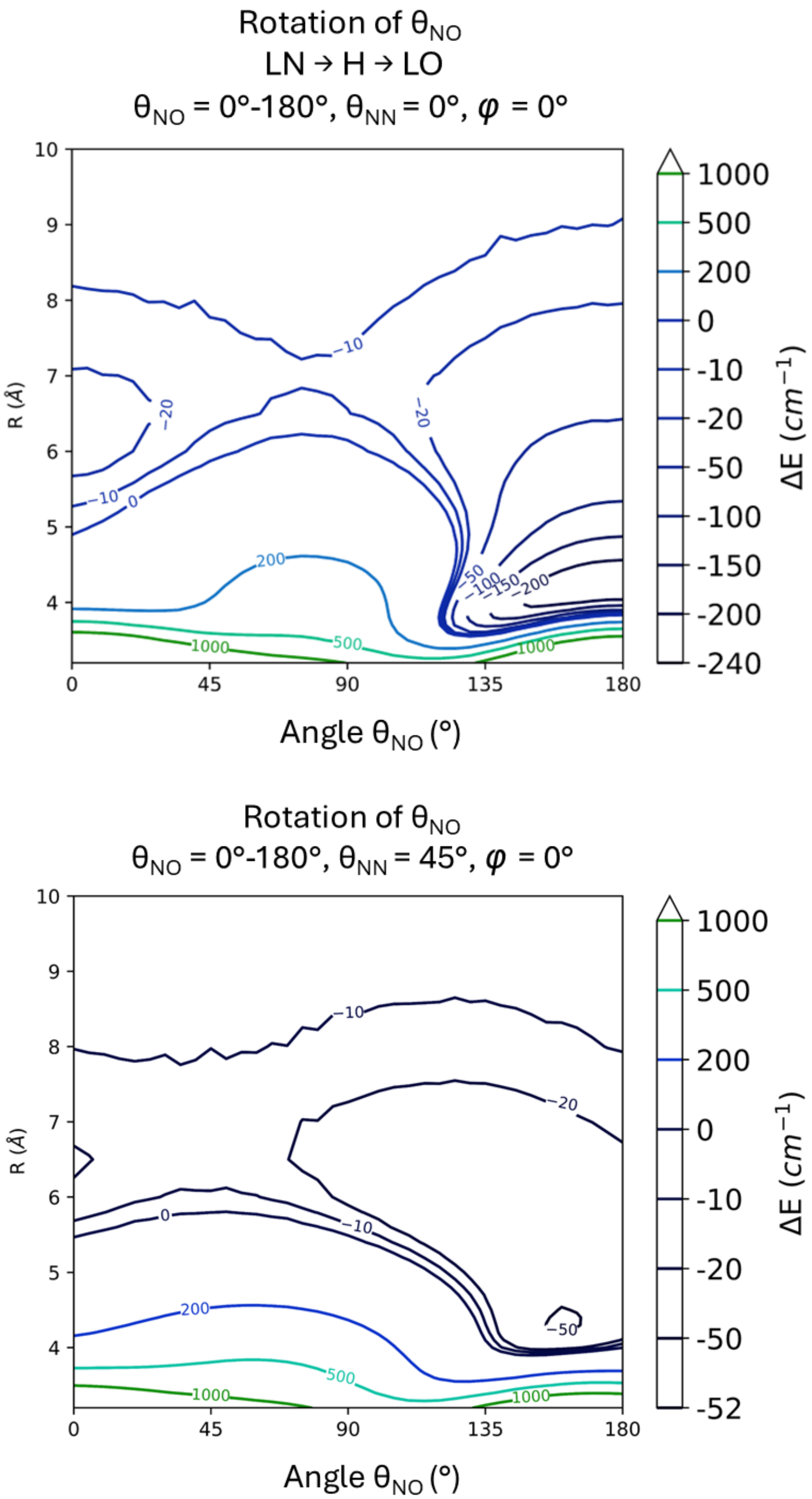

Figure S23: Contour plots of the calculated PES for the $NO(A^2\Sigma^+)$+$N_2(X^1\Sigma^+)$ for rotation of the Θ_{NO} angle. Fixed angles are given for each plot.

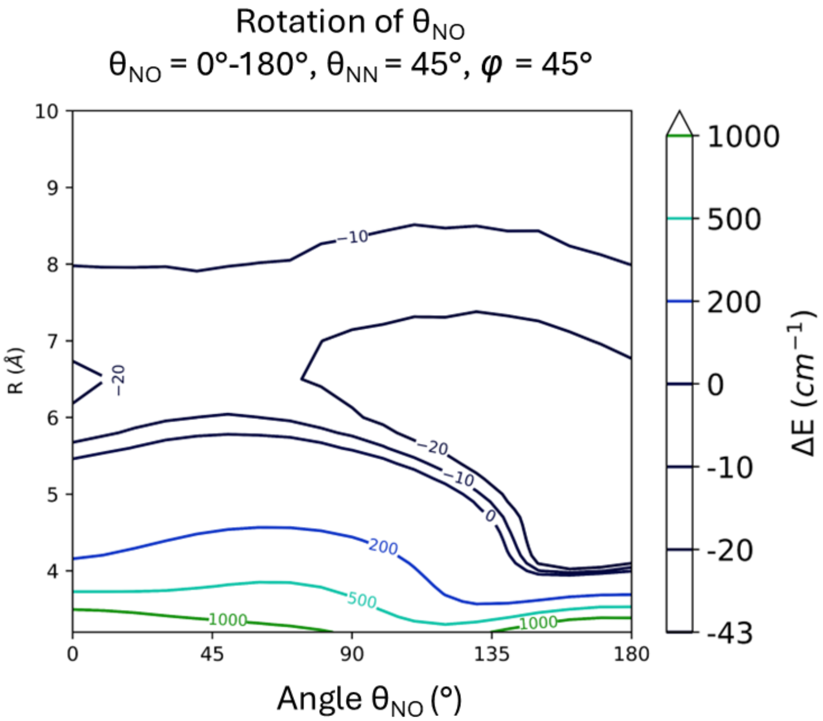

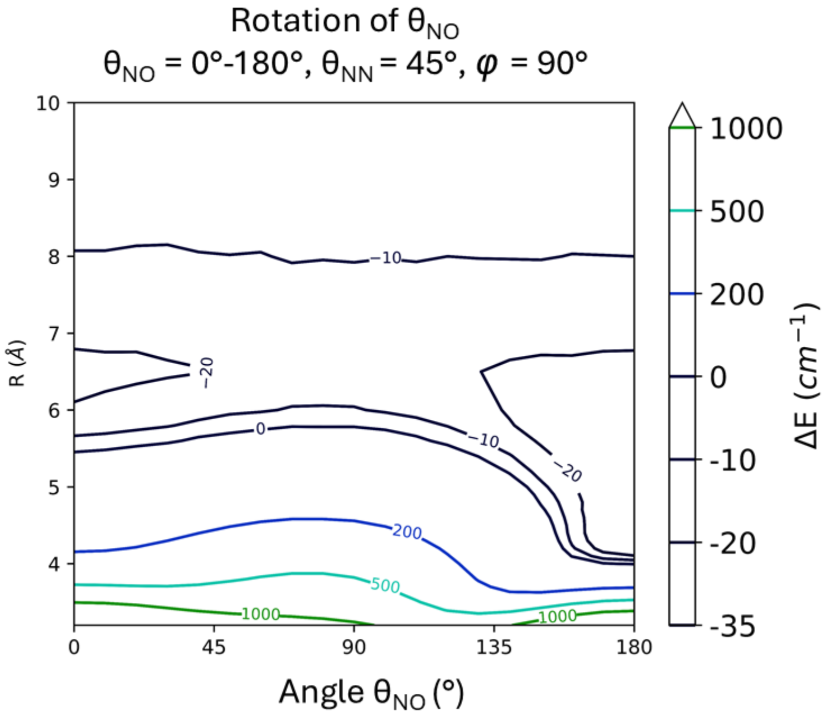

Figure S24: Contour plots of the calculated PES for the $NO(A^2\Sigma^+)+N_2(X^1\Sigma^+)$ for rotation of the Θ_{NO} angle. Fixed angles are given for each plot.

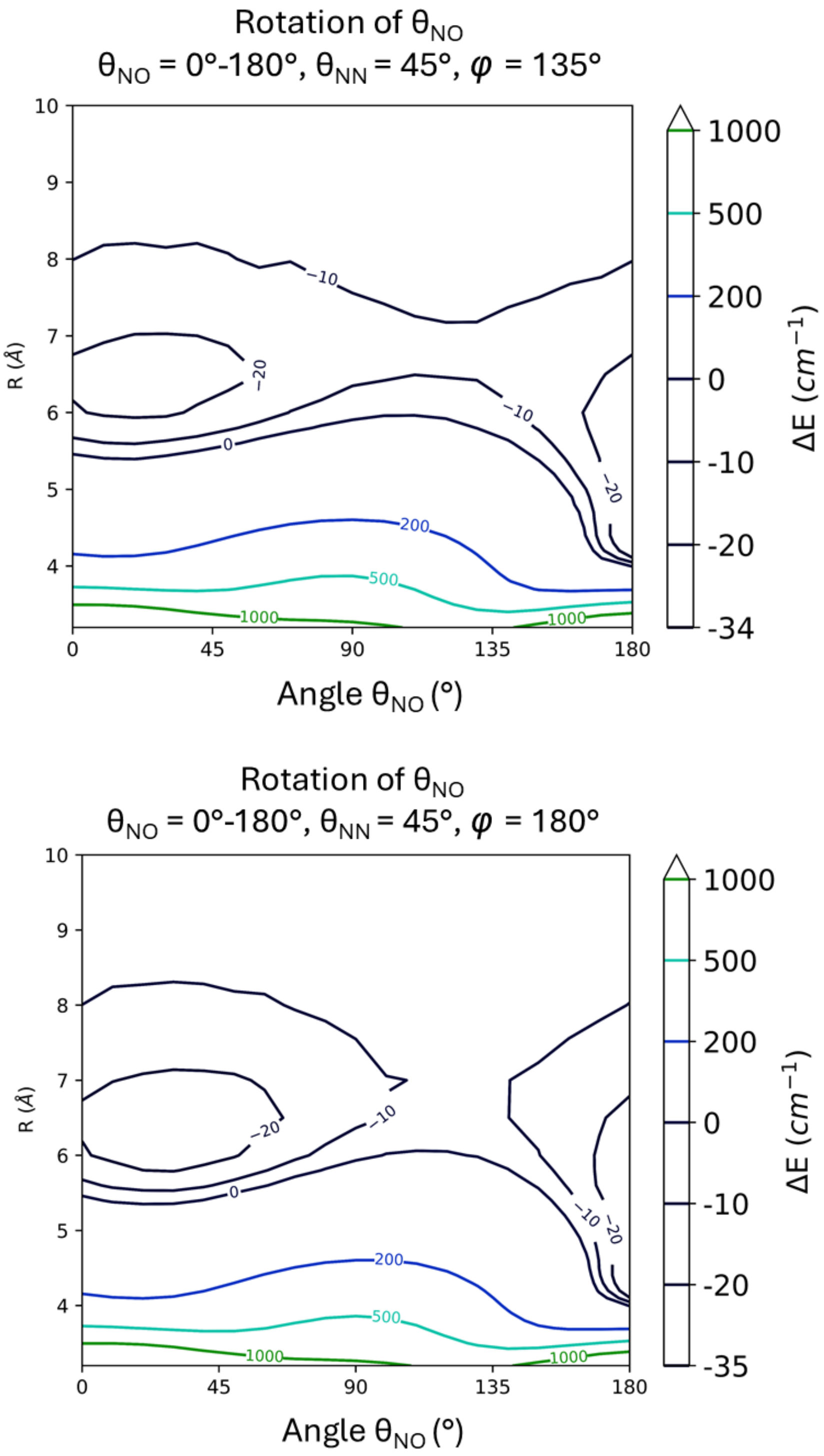

Figure S25: Contour plots of the calculated PES for the $NO(A^2\Sigma^+)$+$N_2(X^1\Sigma^+)$ for rotation of the Θ_{NO} angle. Fixed angles are given for each plot.

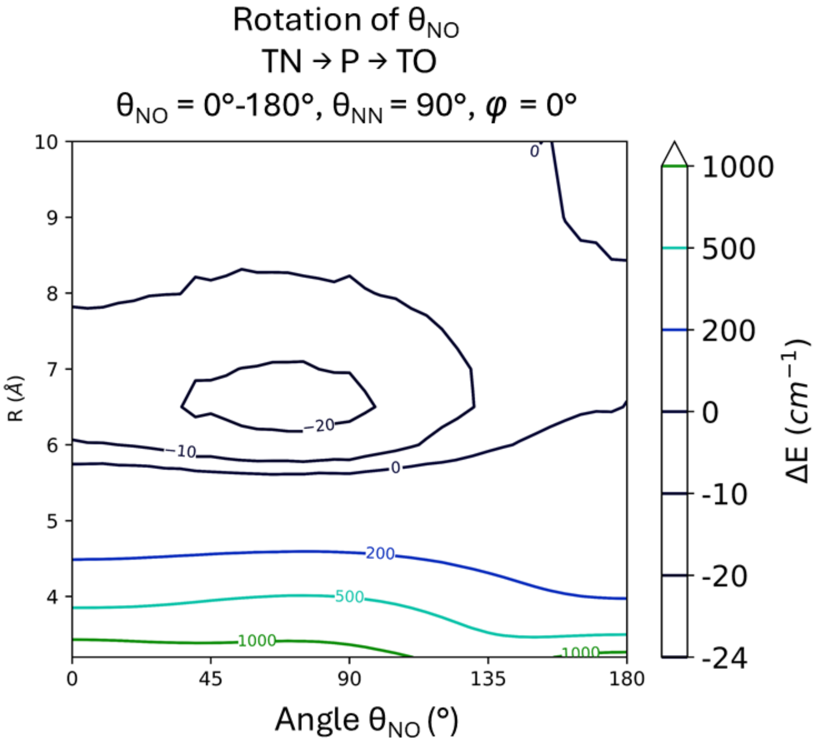

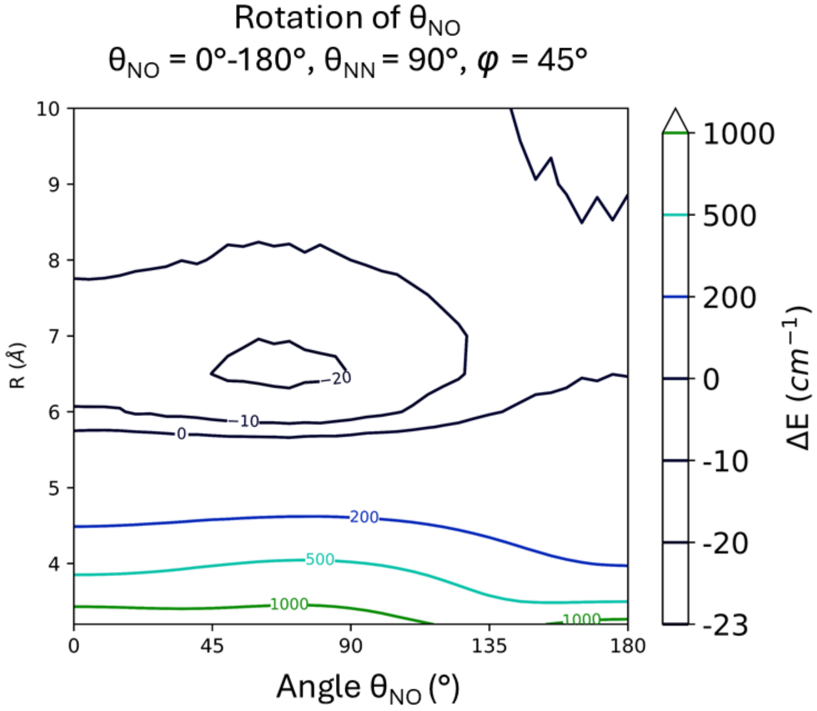

Figure S26: Contour plots of the calculated PES for the $NO(A^2\Sigma^+)$+$N_2(X^1\Sigma^+)$ for rotation of the Θ_{NO} angle. Fixed angles are given for each plot.

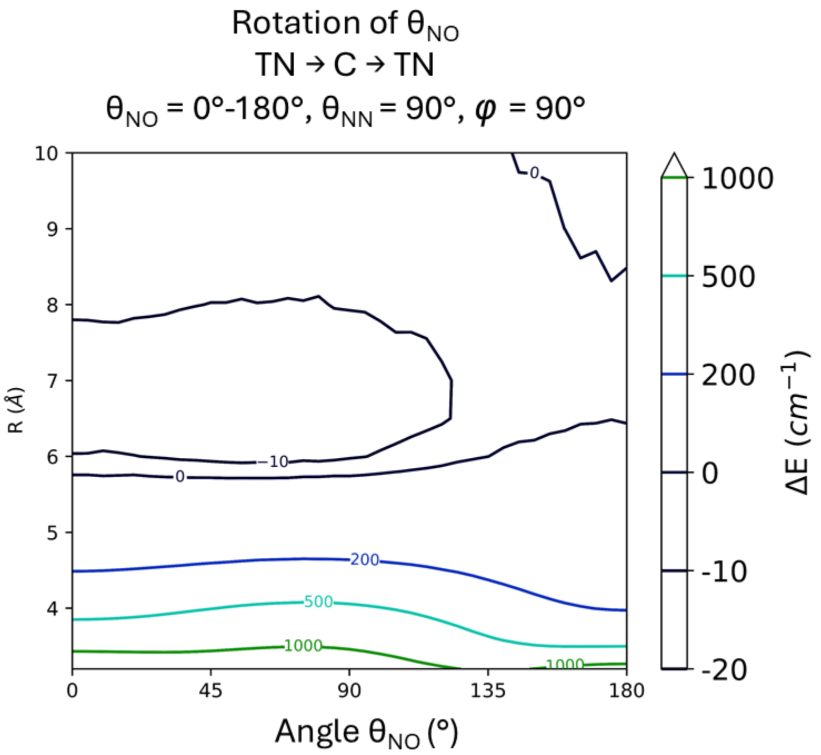

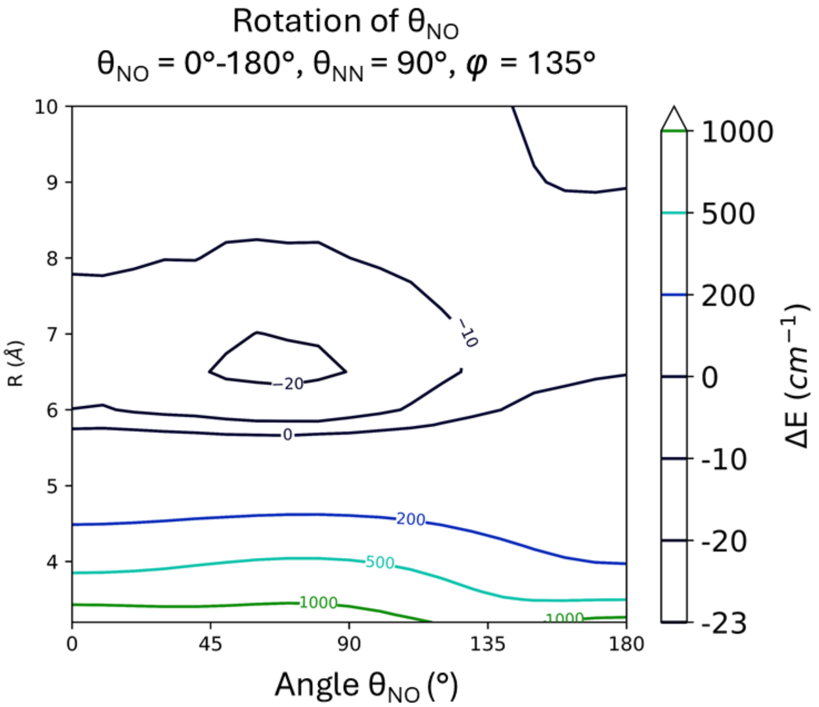

Figure S27: Contour plots of the calculated PES for the $NO(A^2\Sigma^+)+N_2(X^1\Sigma^+)$ for rotation of the Θ_{NO} angle. Fixed angles are given for each plot.

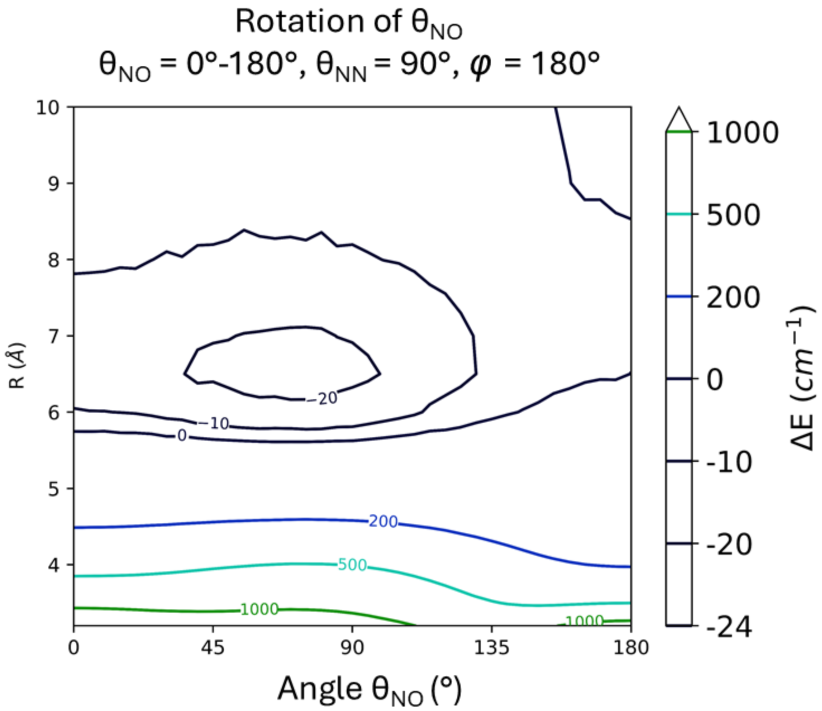

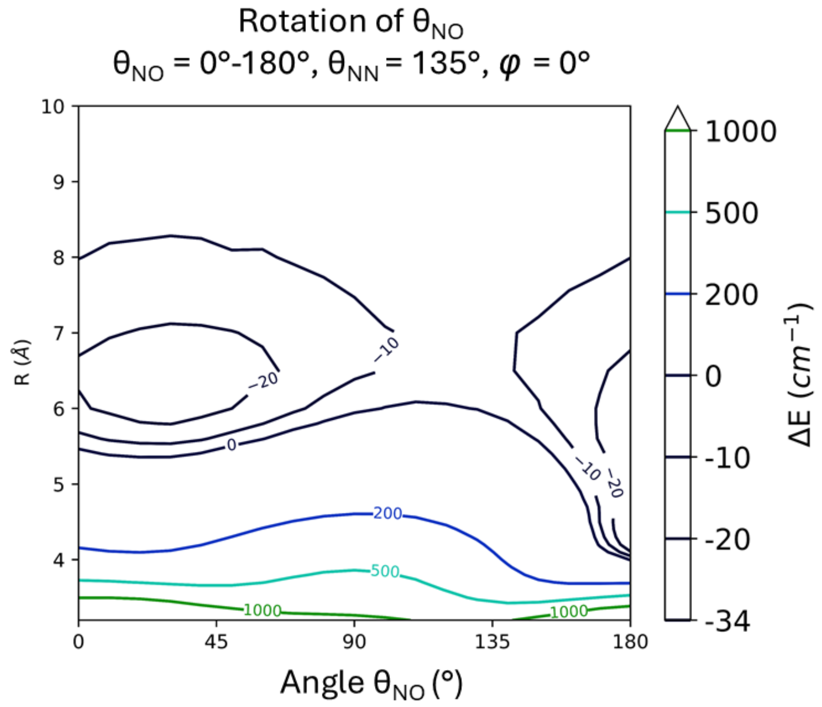

Figure S28: Contour plots of the calculated PES for the $NO(A^2\Sigma^+)+N_2(X^1\Sigma^+)$ for rotation of the Θ_{NO} angle. Fixed angles are given for each plot.

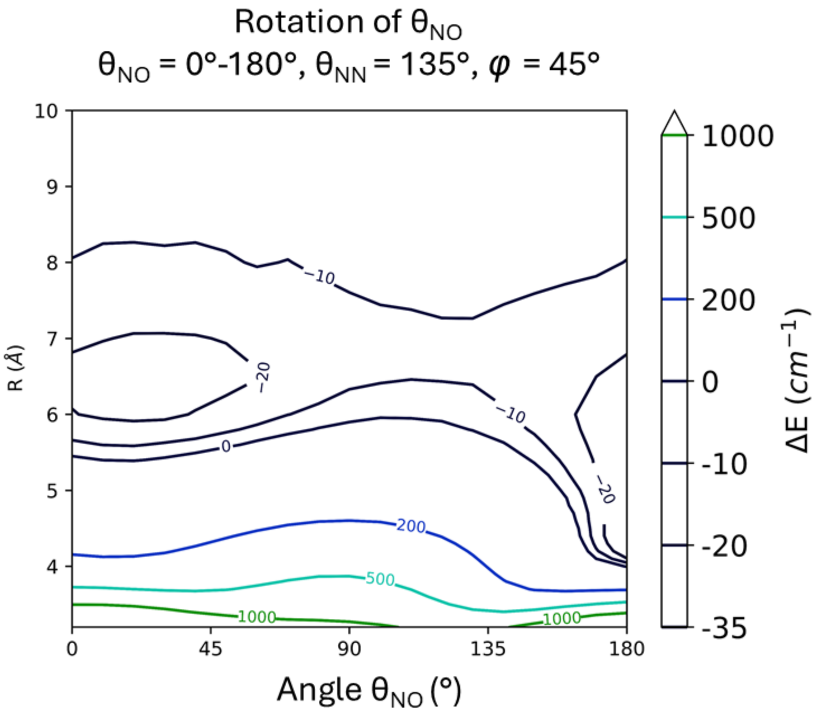

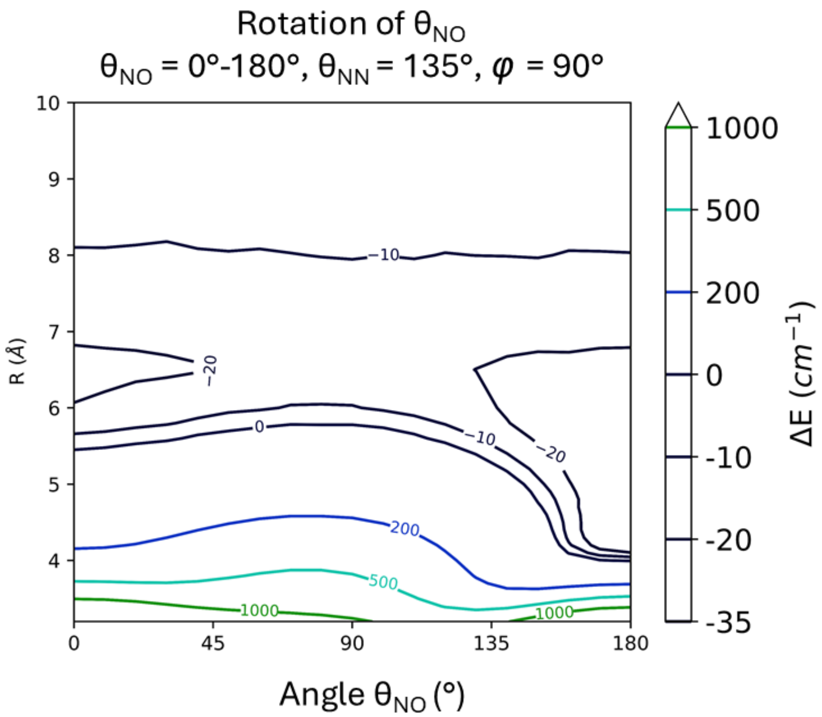

Figure S29: Contour plots of the calculated PES for the $NO(A^2\Sigma^+)+N_2(X^1\Sigma^+)$ for rotation of the Θ_{NO} angle. Fixed angles are given for each plot.

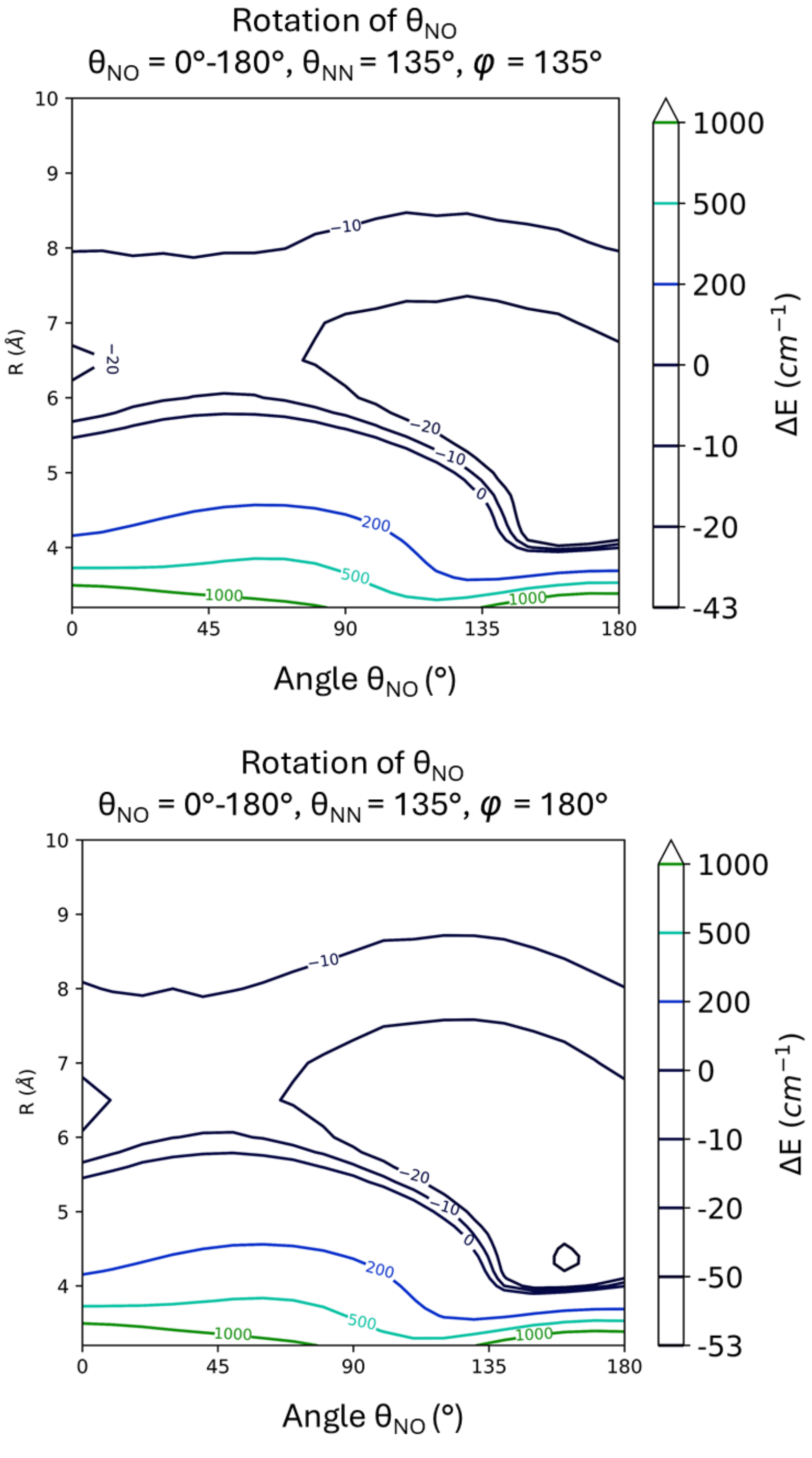

Figure S30: Contour plots of the calculated PES for the $NO(A^2\Sigma^+)+N_2(X^1\Sigma^+)$ for rotation of the Θ_{NO} angle. Fixed angles are given for each plot.

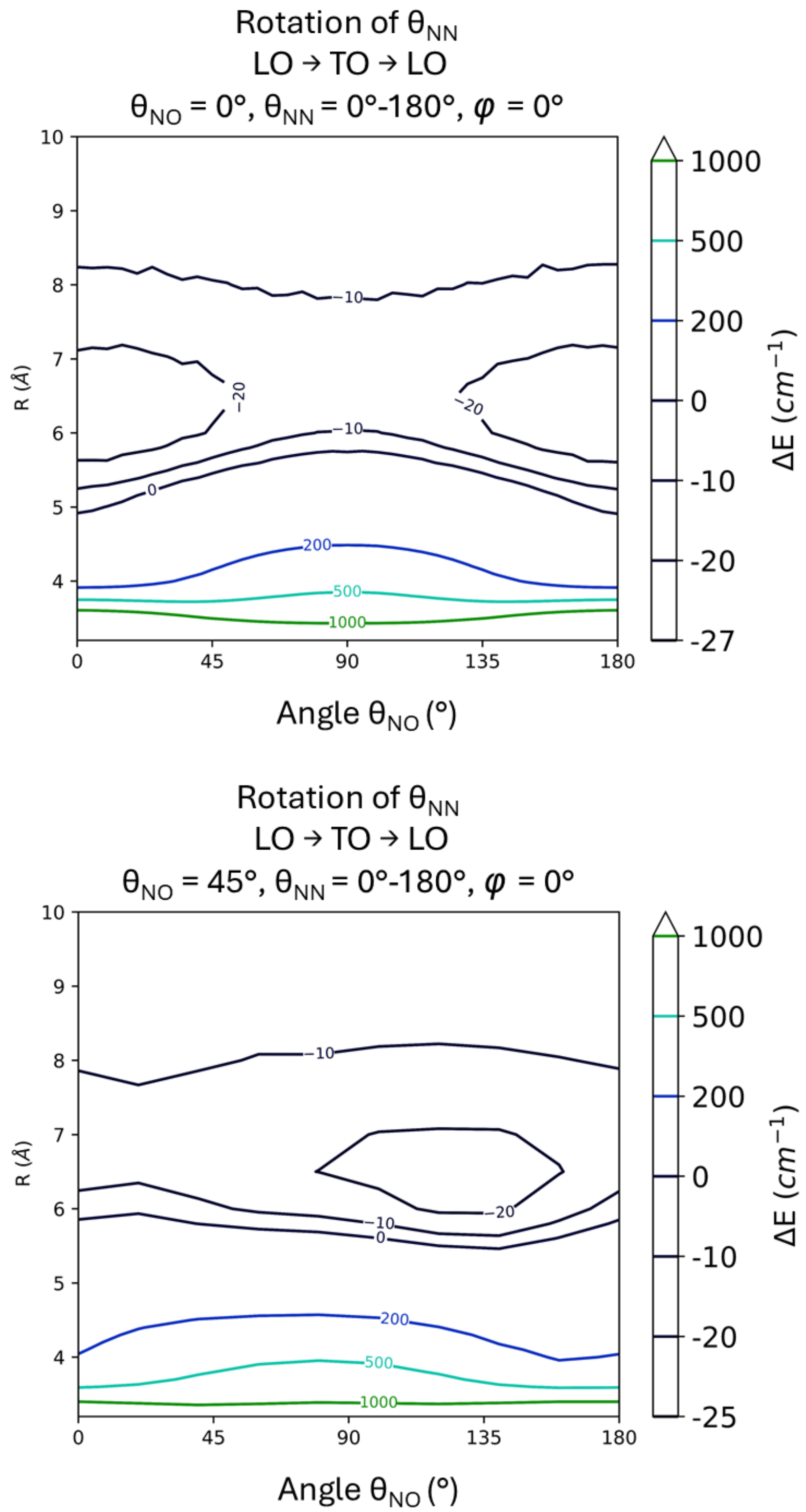

Figure S31: Contour plots of the calculated PES for the $NO(A^2\Sigma^+)+N_2(X^1\Sigma^+)$ for rotation of the Θ_{NN} angle. Fixed angles are given for each plot.

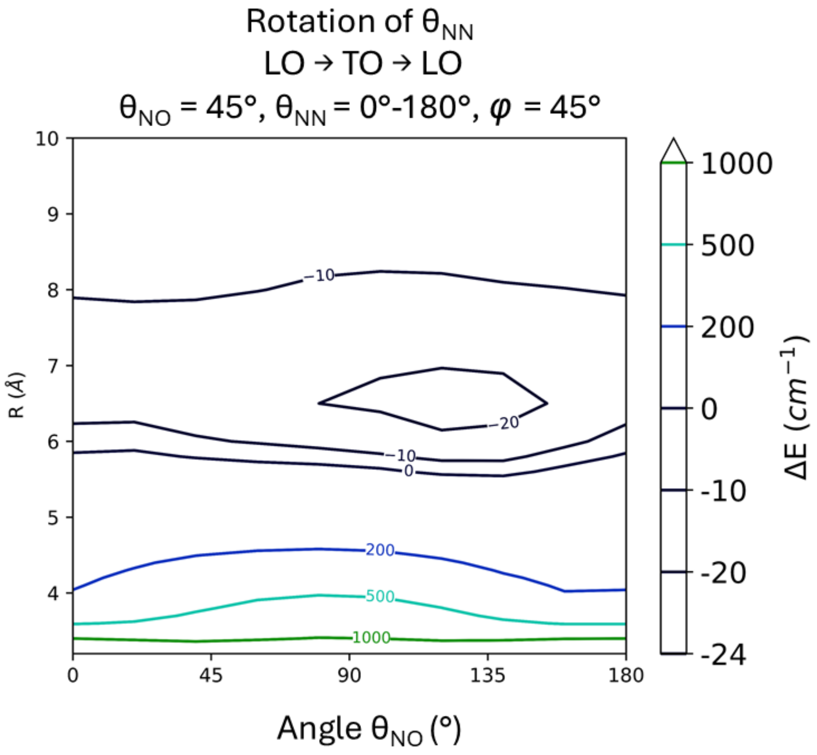

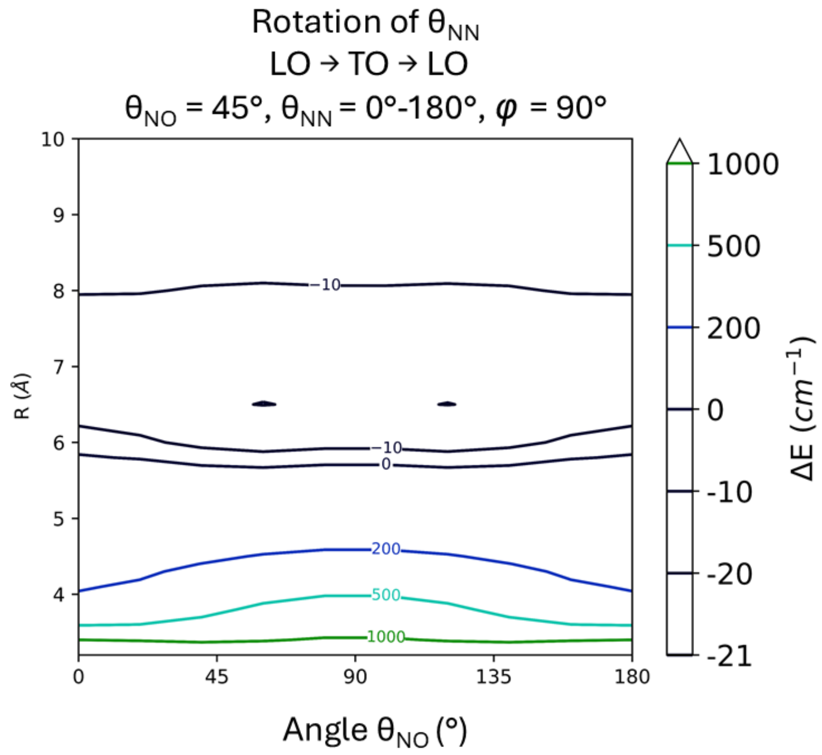

Figure S32: Contour plots of the calculated PES for the $NO(A^2\Sigma^+)+N_2(X^1\Sigma^+)$ for rotation of the Θ_{NN} angle. Fixed angles are given for each plot.

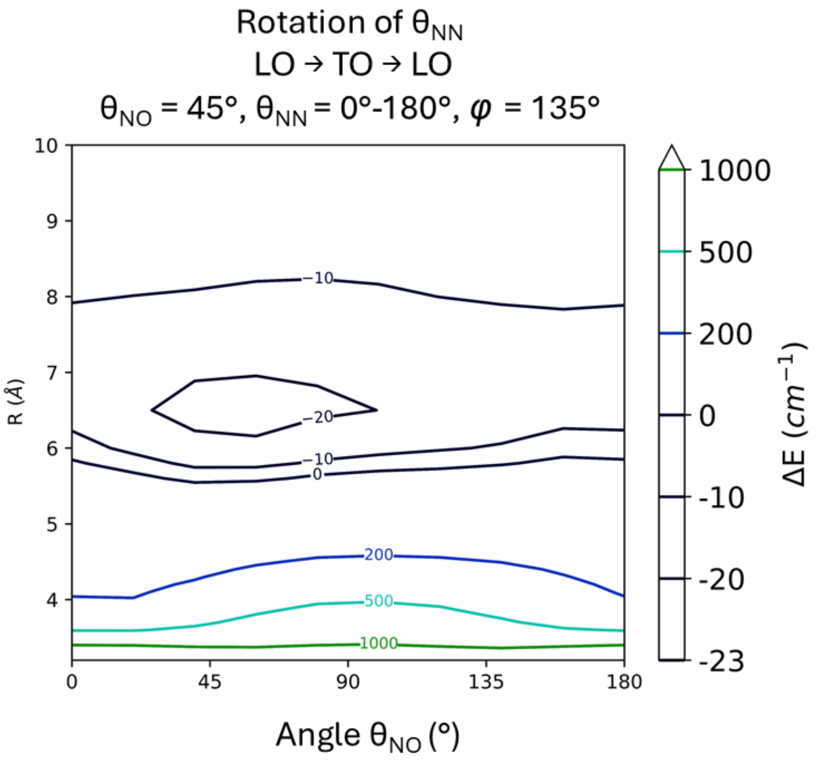

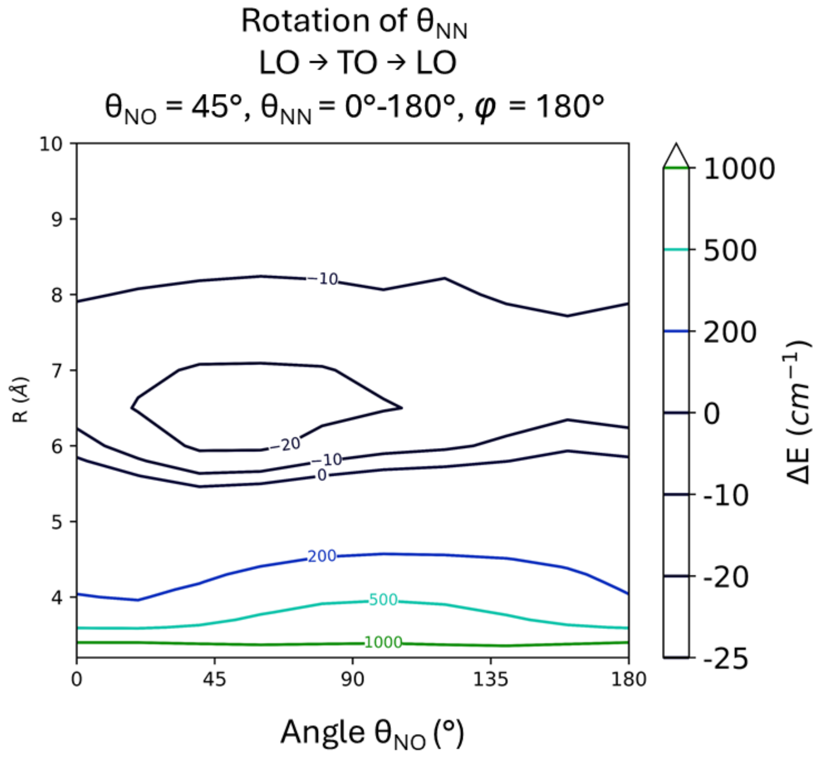

Figure S33: Contour plots of the calculated PES for the $NO(A^2\Sigma^+)+N_2(X^1\Sigma^+)$ for rotation of the Θ_{NN} angle. Fixed angles are given for each plot.

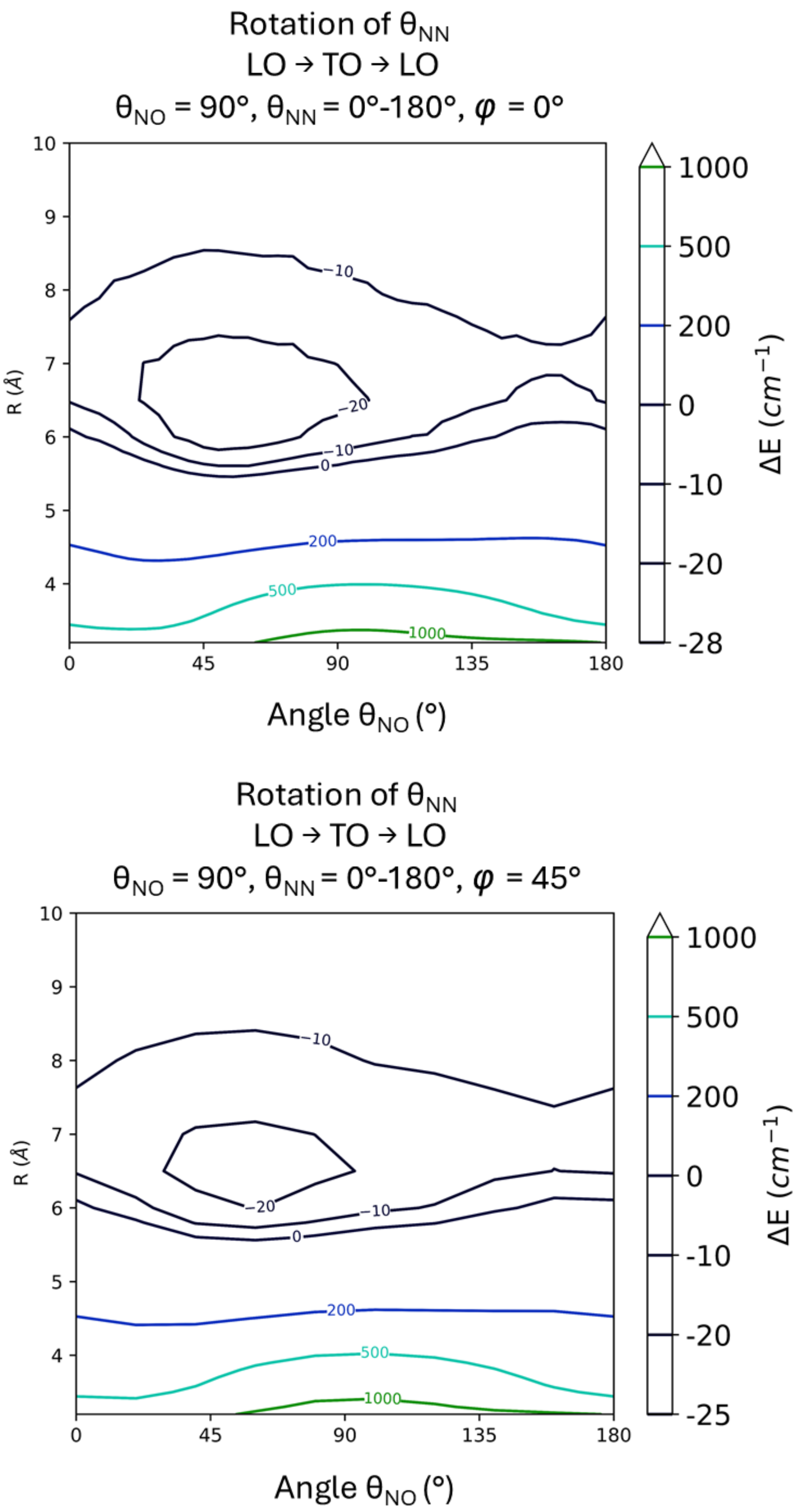

Figure S34: Contour plots of the calculated PES for the $NO(A^2\Sigma^+)+N_2(X^1\Sigma^+)$ for rotation of the Θ_{NN} angle. Fixed angles are given for each plot.

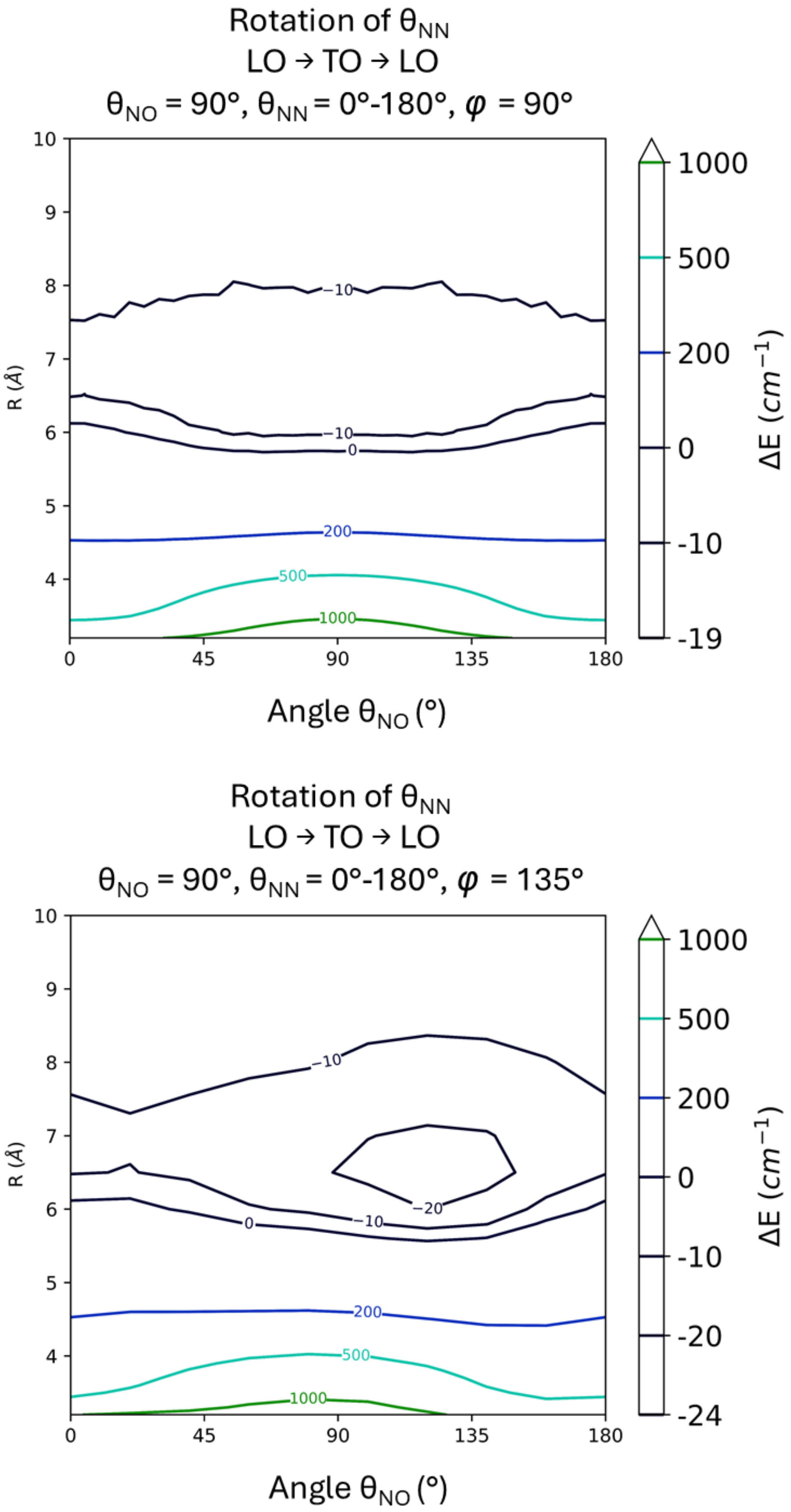

Figure S35: Contour plots of the calculated PES for the $NO(A^2\Sigma^+)$+$N_2(X^1\Sigma^+)$ for rotation of the Θ_{NN} angle. Fixed angles are given for each plot.

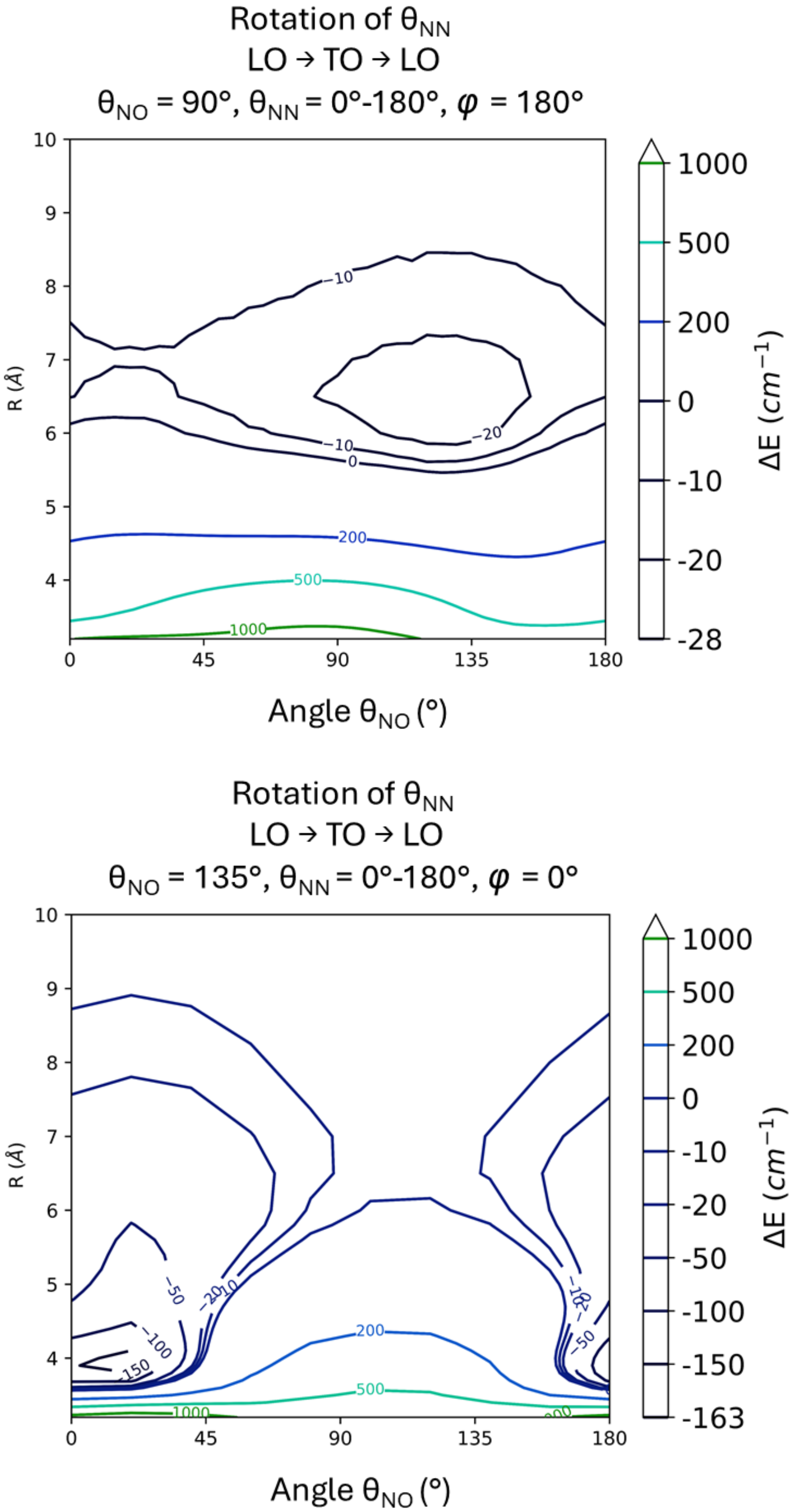

Figure S36: Contour plots of the calculated PES for the $NO(A^2\Sigma^+)+N_2(X^1\Sigma^+)$ for rotation of the Θ_{NN} angle. Fixed angles are given for each plot.

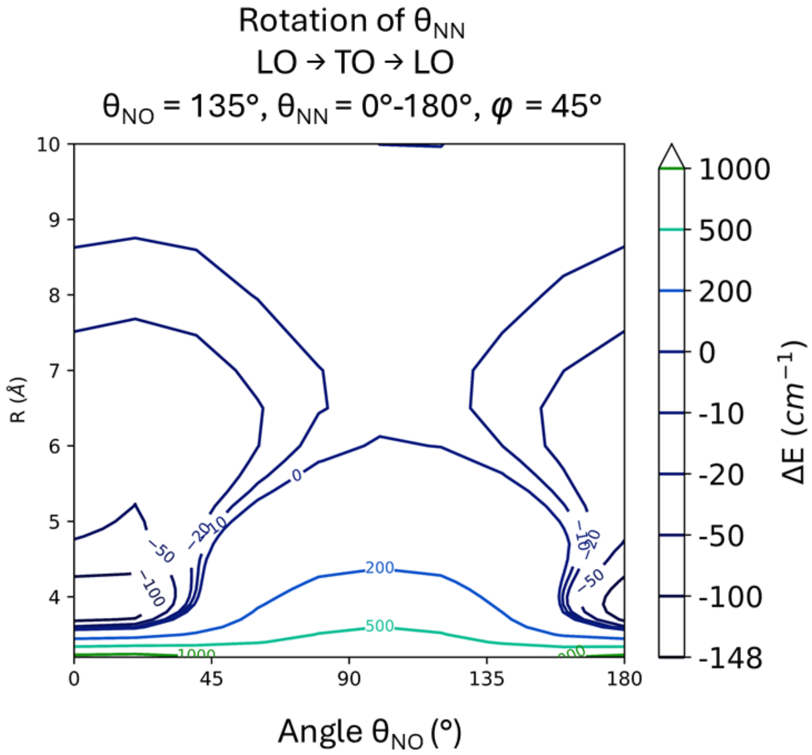

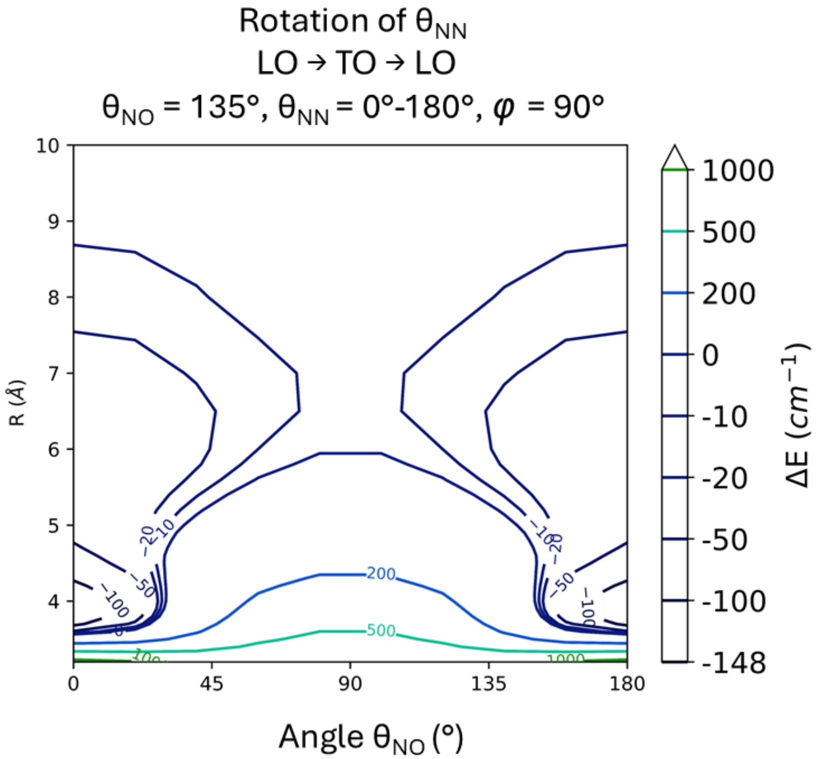

Figure S37: Contour plots of the calculated PES for the $NO(A^2\Sigma^+)+N_2(X^1\Sigma^+)$ for rotation of the Θ_{NN} angle. Fixed angles are given for each plot.

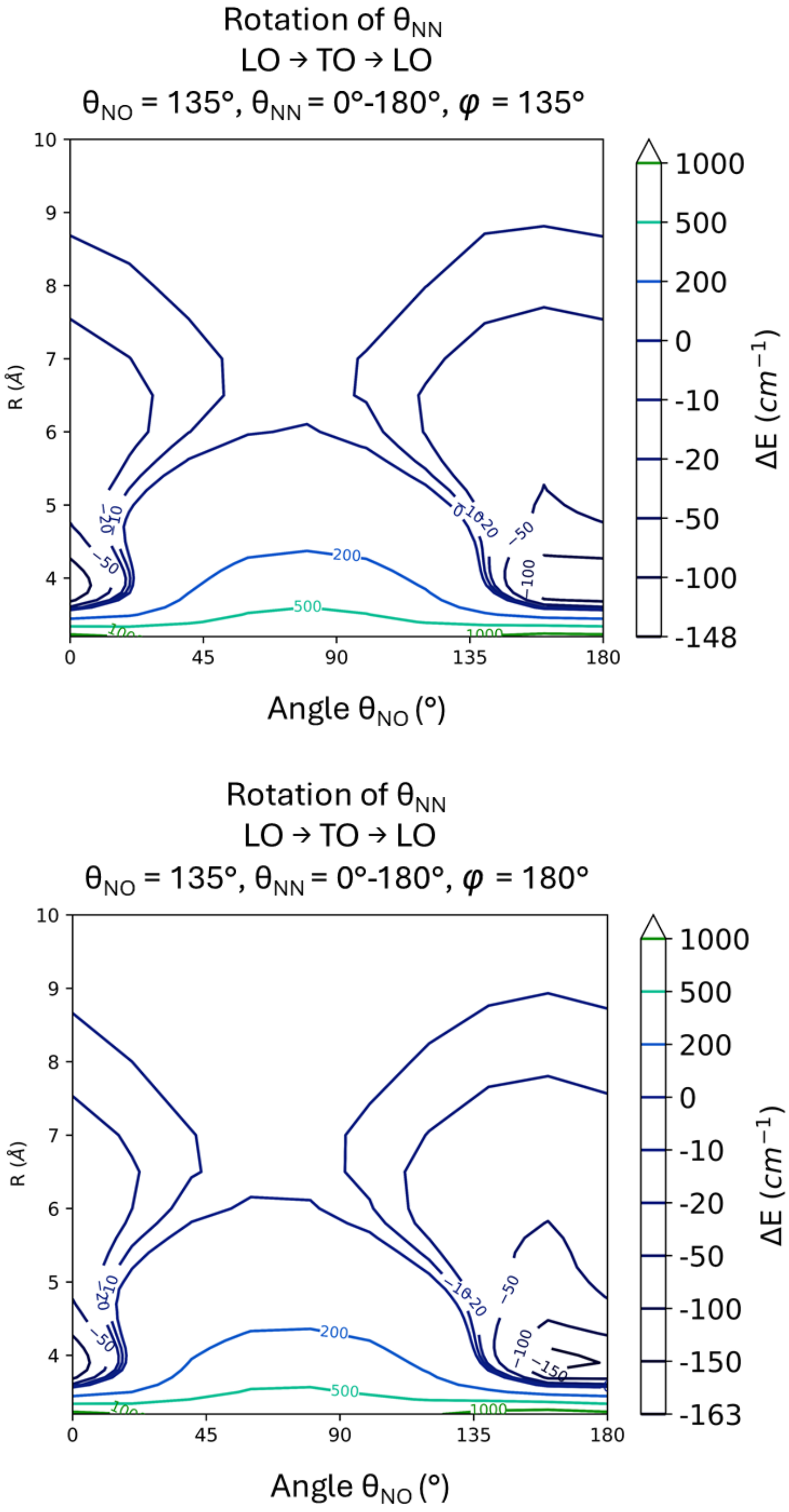

Figure S38: Contour plots of the calculated PES for the $NO(A^2\Sigma^+)$+$N_2(X^1\Sigma^+)$ for rotation of the Θ_{NN} angle. Fixed angles are given for each plot.

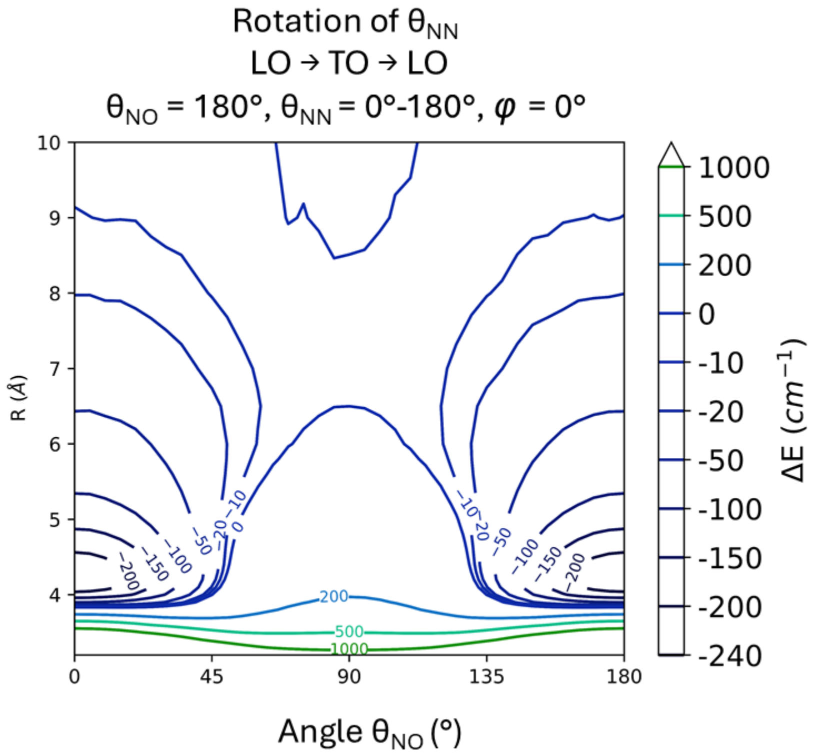

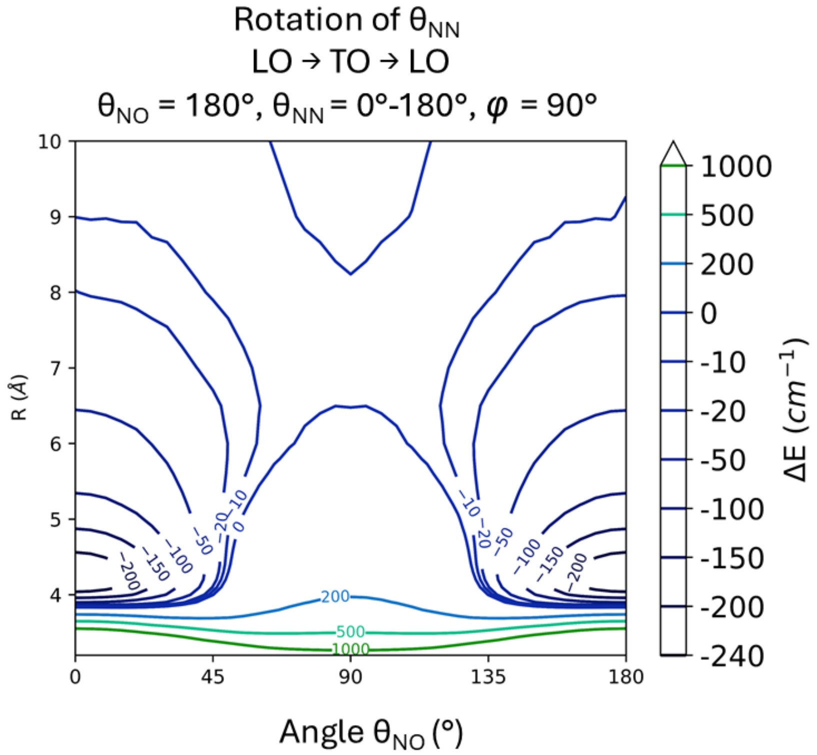

Figure S39: Contour plots of the calculated PES for the $NO(A^2\Sigma^+)+N_2(X^1\Sigma^+)$ for rotation of the Θ_{NN} angle. Fixed angles are given for each plot.

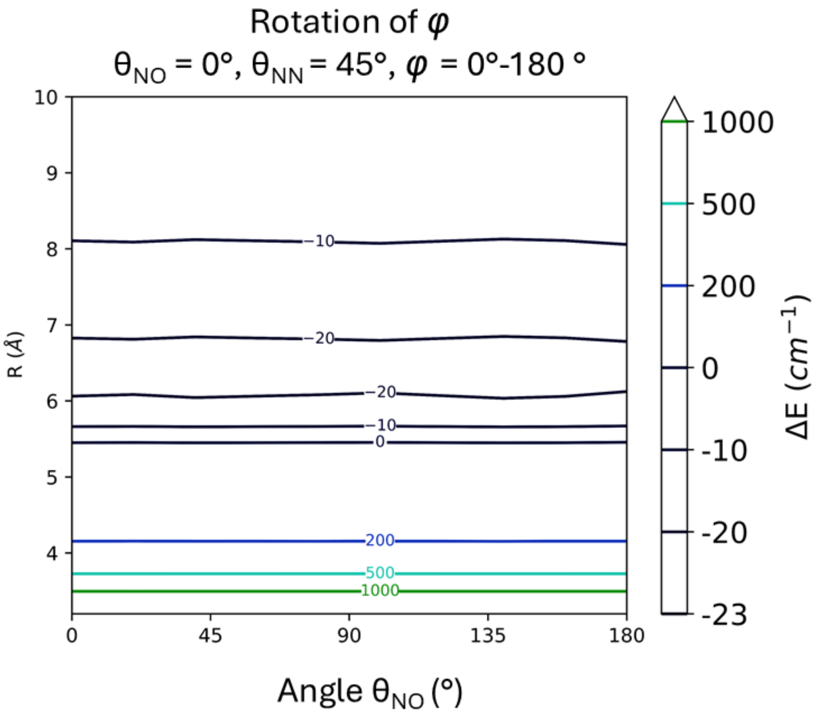

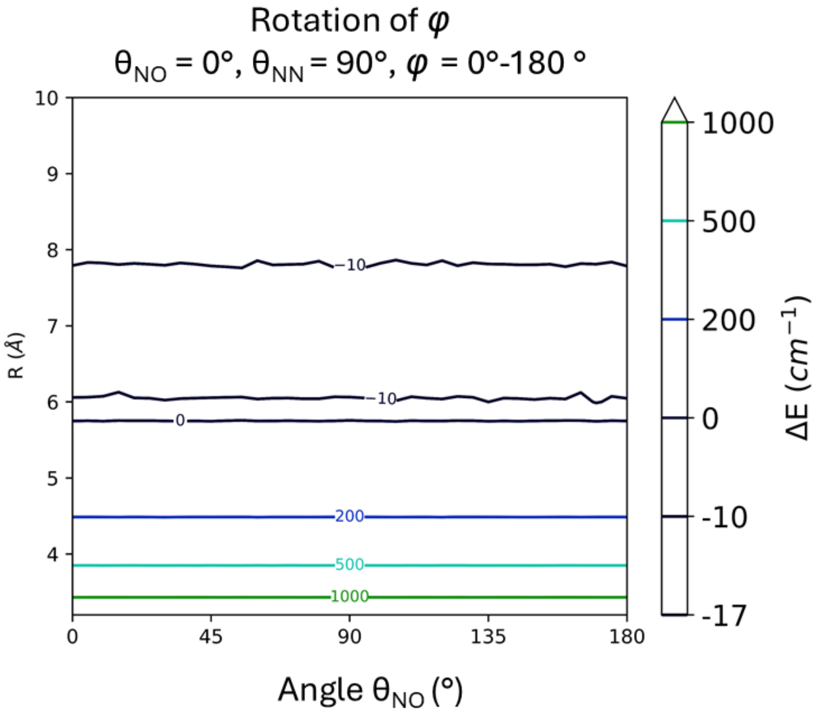

Figure S40: Contour plots of the calculated PES for the $NO(A^2\Sigma^+)+N_2(X^1\Sigma^+)$ for rotation of the ψ angle. Fixed angles are given for each plot.

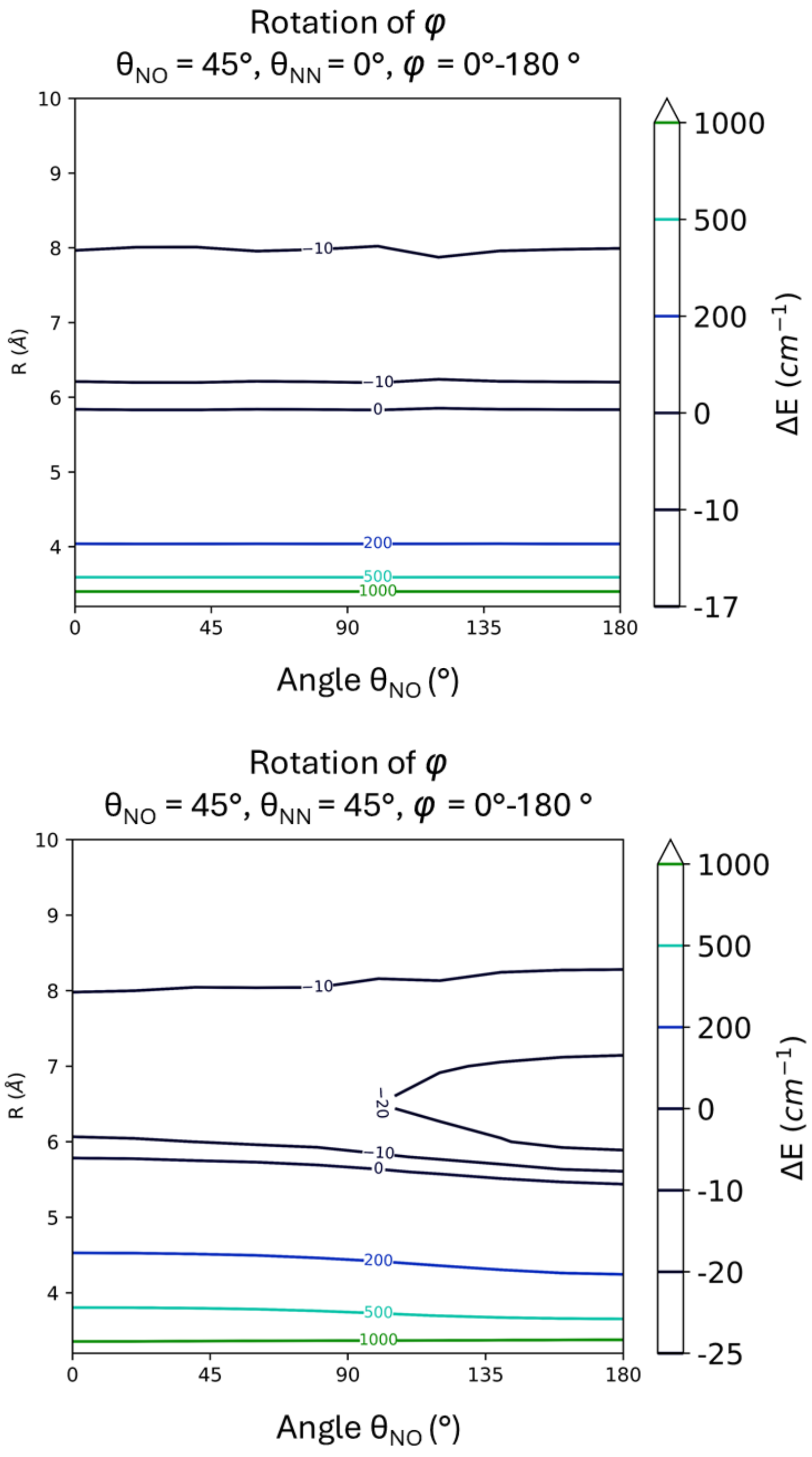

Figure S41: Contour plots of the calculated PES for the $NO(A^2\Sigma^+)+N_2(X^1\Sigma^+)$ for rotation of the ψ angle. Fixed angles are given for each plot.

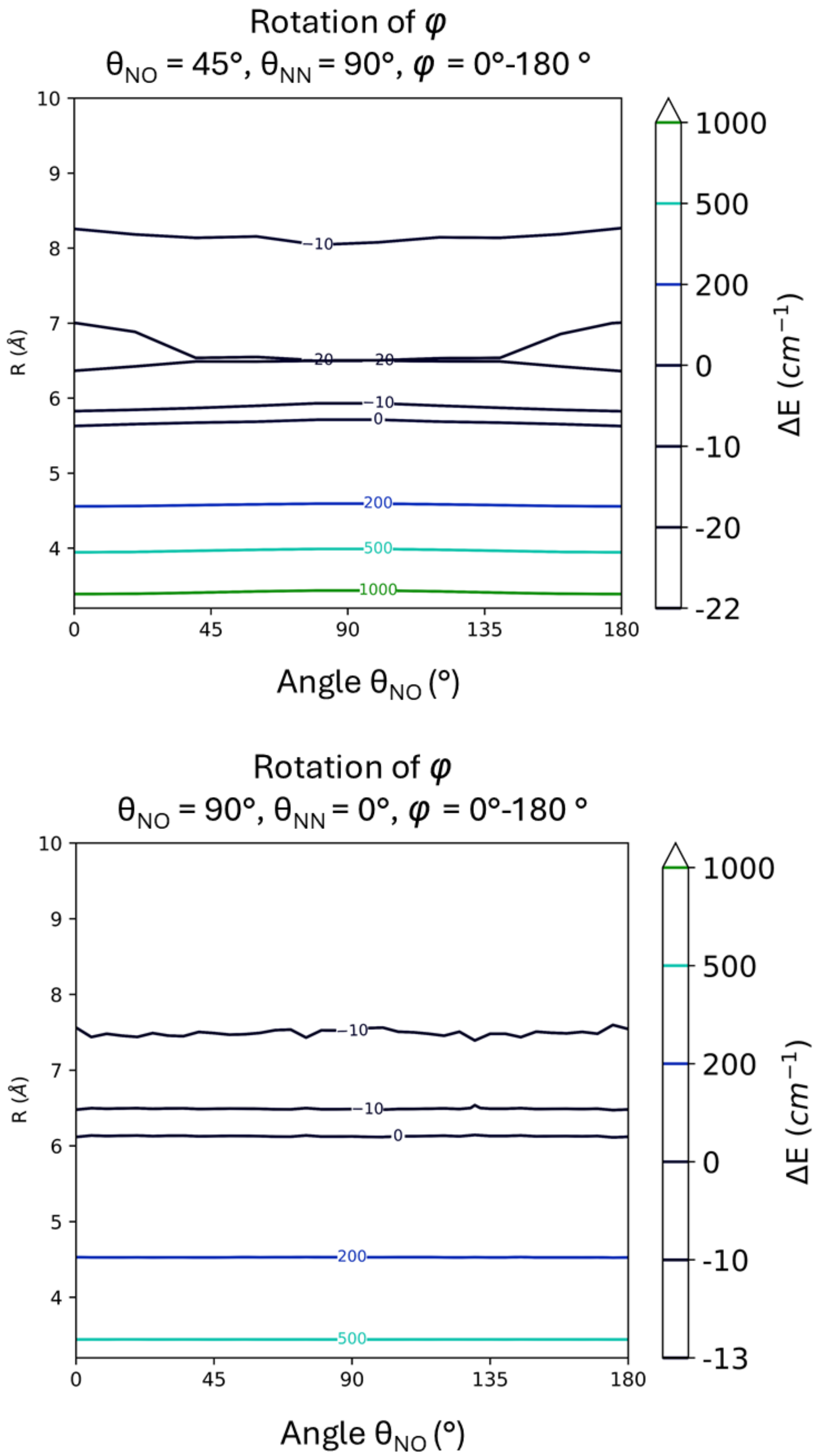

Figure S42: Contour plots of the calculated PES for the $NO(A^2\Sigma^+)$+$N_2(X^1\Sigma^+)$ for rotation of the ψ angle. Fixed angles are given for each plot.

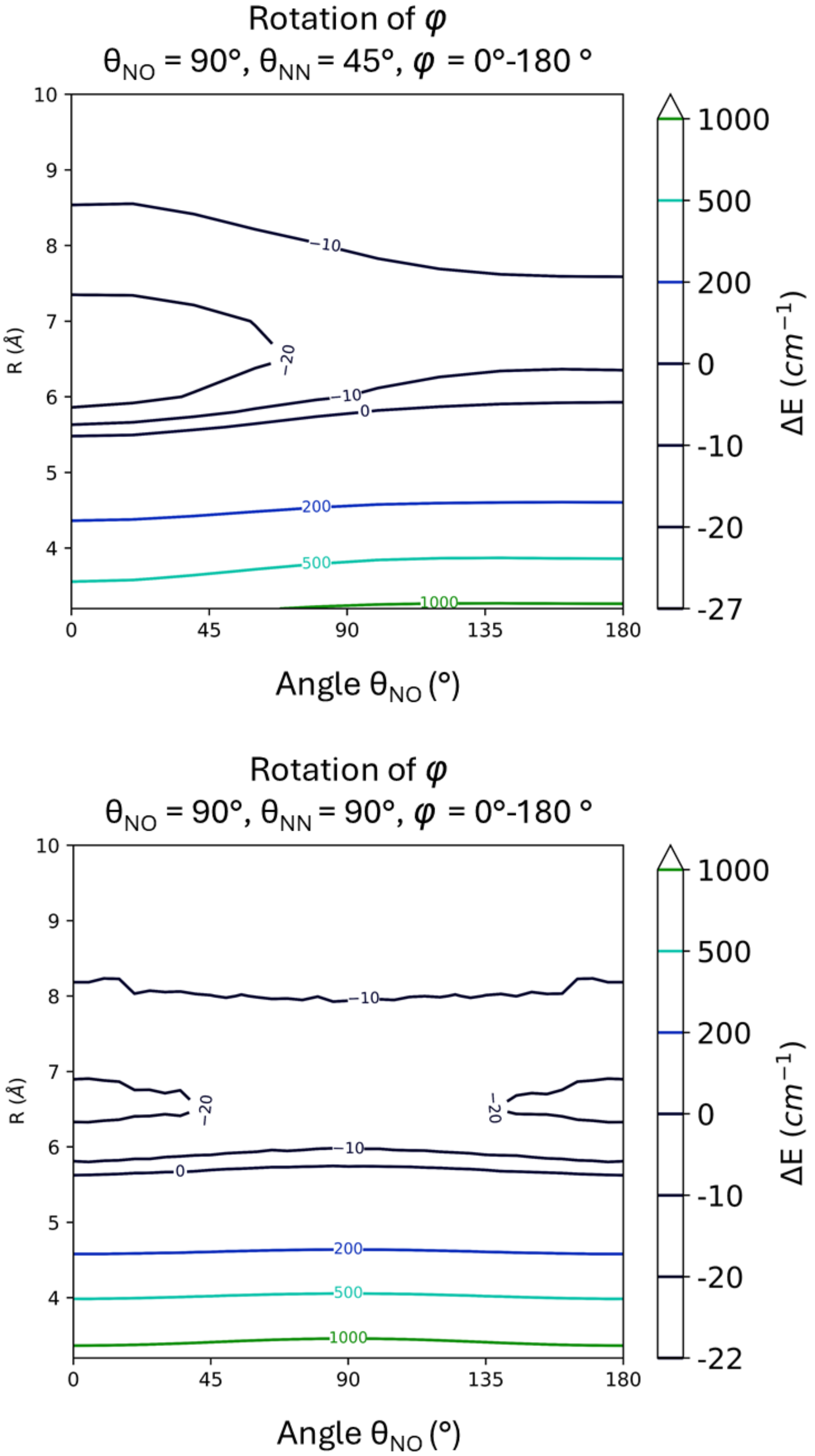

Figure S43: Contour plots of the calculated PES for the $NO(A^2\Sigma^+)$+$N_2(X^1\Sigma^+)$ for rotation of the ψ angle. Fixed angles are given for each plot.

References

[1] Boys, S.; Bernardi, F. The calculation of small molecular interactions by the differences of separate total energies. Some procedures with reduced errors. *Molecular Physics* **1970**, *19*, 553–566.
